# Methylthio-alkane reductases use nitrogenase metalloclusters for carbon–sulfur bond cleavage

**DOI:** 10.1038/s41929-025-01426-2

**Published:** 2025-10-23

**Authors:** Ana Lago-Maciel, Jéssica C. Soares, Jan Zarzycki, Charles J. Buchanan, Tristan Reif-Trauttmansdorff, Frederik V. Schmidt, Stefano Lometto, Nicole Paczia, Jan M. Schuller, D. Flemming Hansen, Gabriella T. Heller, Simone Prinz, Georg K. A. Hochberg, Antonio J. Pierik, Johannes G. Rebelein

**Affiliations:** 1 Microbial Metalloenzymes Research Group, Max Planck Institute for Terrestrial Microbiology, Marburg, Germany; 2 Department of Chemistry, RPTU University Kaiserslautern-Landau, Kaiserslautern, Germany; 3 Department of Biochemistry and Synthetic Metabolism, Max Planck Institute for Terrestrial Microbiology, Marburg, Germany; 4 Evolutionary Biochemistry Group, Max Planck Institute for Terrestrial Microbiology, Marburg, Germany; 5 Department of Structural and Molecular Biology, Division of Biosciences, University College London, London, UK; 6 Department of Chemistry, Philipps–University Marburg, Marburg, Germany; 7 Metabolomics Core Facility, Max Planck Institute for Terrestrial Microbiology, Marburg, Germany; 8 Center for Synthetic Microbiology (SYNMIKRO), Philipps–University Marburg, Marburg, Germany; 9 The Francis Crick Institute, London, UK; 10 Central Electron Microscopy Facility, Max Planck Institute of Biophysics, Frankfurt am Main, Germany

**Keywords:** Biocatalysis, Cryoelectron microscopy, Oxidoreductases, Carbon cycle

## Abstract

Methylthio-alkane reductases convert methylated sulfur compounds to methanethiol and small hydrocarbons, a process with important environmental and biotechnological implications. These enzymes are classified as nitrogenase-like enzymes, despite lacking the ability to convert dinitrogen to ammonia, raising fundamental questions about the factors controlling their activity and specificity. Here we present the molecular structure of the methylthio-alkane reductase, which reveals large metalloclusters, including the P-cluster and the [Fe_8_S_9_C]-cluster, previously found only in nitrogenases. Our findings suggest that distinct metallocluster coordination, surroundings and substrate channels determine the activity of these related metalloenzymes. This study provides new insights into nitrogen fixation, sulfur-compound reduction and hydrocarbon production. We also shed light on the evolutionary history of P-cluster and [Fe_8_S_9_C]-cluster-containing reductases emerging before nitrogenases.

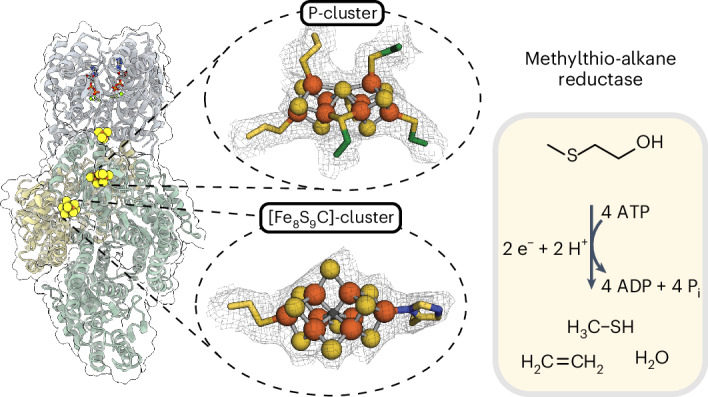

## Abstract (web summary)

Methylthio-alkane reductases are recently discovered enzymes that can produce methanethiol and small hydrocarbons from methylated sulfur compounds. Now the cryo-EM structure of a methylthio-alkane reductase complex is solved, revealing large metalloclusters previously observed only within nitrogenases.

## Main

Sulfur is an essential element in amino acids, vitamins and protein cofactors. Bacteria assimilate inorganic sulfur through the uptake and reduction of sulfate^1^. However, under sulfate unavailability, bacteria scavenge organic sulfur via methionine-salvage pathways by recycling metabolic by-products to methionine^2,3^. *Rhodospirillum rubrum* utilizes an anaerobic methionine-salvage pathway to convert (2-methylthio)ethanol (MT-EtOH) to methanethiol, direct precursor to methionine, via methylthio-alkane reductase^4,5^. In vivo, this enzyme produces methanethiol from several volatile organic sulfur compounds (VOSCs) besides MT-EtOH, including dimethyl sulfide (DMS) and ethyl methyl sulfide (EMS), releasing in a 1:1 stoichiometry ethylene (C_2_H_4_), methane (CH_4_) or ethane (C_2_H_6_), respectively^4,5^. The release of these small hydrocarbons, which are potent greenhouse gases, to the atmosphere can exacerbate the climate crisis. However, these hydrocarbons also offer a biological, sustainable alternative to fossil fuel-based products, like natural gas and plastic precursors. Specifically in the field of renewable plastic production, the methylthio-alkane reductase stands out as a biogenic source of C_2_H_4_ (ref. ^6^). Unlike all other C_2_H_4_-forming enzymes, including the 1-aminocyclopropane-1-carboxylic acid oxidase (ACCO), the ethylene-forming enzyme (EFE) and the NADH:Fe(III)EDTA oxidoreductase, the methylthio-alkane reductase is oxygen independent, requires simple substrates and does not release carbon dioxide (CO_2_) as a side product^6^. This makes this enzyme an exceptional option for the production of this important chemical building block, currently commercially derived exclusively from fossil fuels. Moreover, methylthio-alkane reductases explain the observed accumulation of C_2_H_4_ in waterlogged anoxic soils^7^ and, thus, are potentially involved in agricultural yield losses due to inhibitory effects of C_2_H_4_ on plant growth^8^.

**Fig. 1:**
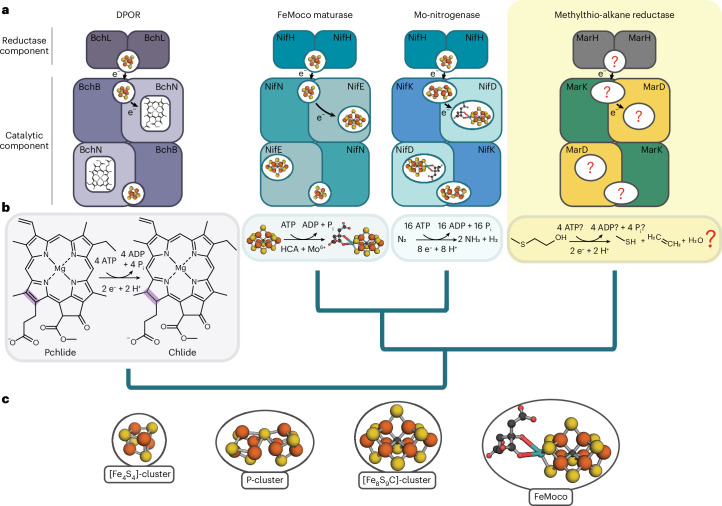
Structural diversity of the nitrogenase and Nfl protein family. **a**, A schematic overview illustrating structures from key nitrogenase and Nfl proteins with their associated metalloclusters responsible for electron transfer and catalysis. Electrons are sequentially transferred through transient interaction of the homodimeric reductase components to the metal cofactors of the heterotetrameric catalytic components. All reductase components harbour an [Fe_4_S_4_]-cluster, which donates electrons to the subunit bridging metallocofactor of the catalytic component. For the DPOR (Bch(NB)_2_) and FeMoco maturase Nif(EN)_2_, this is an [Fe_4_S_4_]-cluster^15,72^, while the Mo-nitrogenase catalytic component (Nif(DK)_2_) harbours a P-cluster ([Fe_8_S_7_]-cluster) as an electron relay to the active site^11^. In DPOR, the substrate protochlorophyllide *a* (Pchlide) sits directly at the active site^72,73^. Nitrogenases harbour more complex metalloclusters in their active sites, such as FeMoco^11^, and Nif(EN)_2_ harbours the [Fe_8_S_9_C]-cluster^15^. The metallocluster composition for the methylthio-alkane reductase as well as the stoichiometry of the reaction was still unresolved. The protein name of each subunit is shown and derived from the corresponding gene name. Genes for each nitrogenase(-like) enzyme are usually organized in an individual operon encoding the subunits of reductase and catalytic components. Not shown here is the very distant homologue Ni^2+^-sirohydrochlorin *a*,*c*-diamide reductase (CfbD), which catalyses the second last step of coenzyme F_430_ biosynthesis, a tetrapyrrole cofactor in methanogens^45^. **b**, Main catalytic reactions. DPOR reduces the C17=C18 double bond of Pchlide to chlorophyllide *a* (Chlide) in the chlorophyll *a* biosynthetic pathway^72,73^. The Nif(EN)_2_ maturase converts the precursor [Fe_8_S_9_C]-cluster into FeMoco by inserting Mo and (*R*)-homocitrate^15^. Mo-nitrogenase reduces protons and N_2_ to NH_3_ and H_2_ (ref. ^11^). The methylthio-alkane reductase is proposed to reduce MT-EtOH to methanethiol and C_2_H_4_ (ref. ^4^). Protein phylogenetic relationships are depicted based on Extended Data Fig. 10. **c**, Legend of the metalloclusters found in nitrogenase and Nfl proteins shown in **a**.

Methylthio-alkane reductases have been phylogenetically classified as nitrogen fixation-like (Nfl) enzymes of the nitrogenase superfamily^4,9^ (Fig. 1). Nitrogenases are the only enzymes known to reduce the stable triple bond of dinitrogen (N_2_) to produce bioavailable ammonia (NH_3_)^10^ and distinguish themselves from Nfl enzymes in their catalytic metal centres^11^. Nfl enzymes such as the dark-operative protochlorophyllide *a* oxidoreductase (DPOR, encoded by *bchLNB*) or the Ni^2+^-sirohydrochlorin *a*,*c*-diamide reductase (encoded by *cfbCD*), involved in carbon–carbon double-bond reductions, contain only [Fe_4_S_4_]-clusters and tetrapyrrole binding sites^12^. By contrast, nitrogenases are known to contain large and complex metalloclusters that allow N_2_ fixation, including the P-cluster, an [Fe_8_S_7_]-cluster and the iron-molybdenum cofactor (FeMoco), a [MoFe_7_S_9_C-(*R*)-homocitrate]-cluster located in the active site of the molybdenum (Mo)-nitrogenase (encoded by *nifHDK*)^11,13,14^ (Fig. 1). FeMoco is assembled by the maturase Nif(EN)_2_, a nitrogenase family member that converts an Fe-only precursor, the [Fe_8_S_9_C]-cluster (L-cluster), into FeMoco by replacing a terminal Fe atom with Mo and attaching (*R*)-homocitrate^15,16^ (Fig. 1). Distinct activities of nitrogenases and Nfl enzymes are attributed to these differences in metallocluster content and, thus, there is great interest to identify the metal centres dictating the carbon–sulfur (C–S) bond reduction activity in methylthio-alkane reductases.

Here, we determined the molecular structure of the methylthio-alkane reductase complex by anaerobic single-particle cryogenic electron microscopy (cryo-EM) yielding a global resolution of 2.75 Å. We found that the catalytic component contains P-clusters and [Fe_8_S_9_C]-clusters, making it the only Nfl enzyme with large nitrogenase cofactors, but unable to reduce N_2_. Our findings suggest that the protein scaffold, rather than the metalloclusters alone, dictates the differing catalytic activities of nitrogenases and methylthio-alkane reductases, advancing our understanding of the origin and evolution of N_2_ fixation.

## Results

### Structural characterization of the methylthio-alkane reductase

**Fig. 2:**
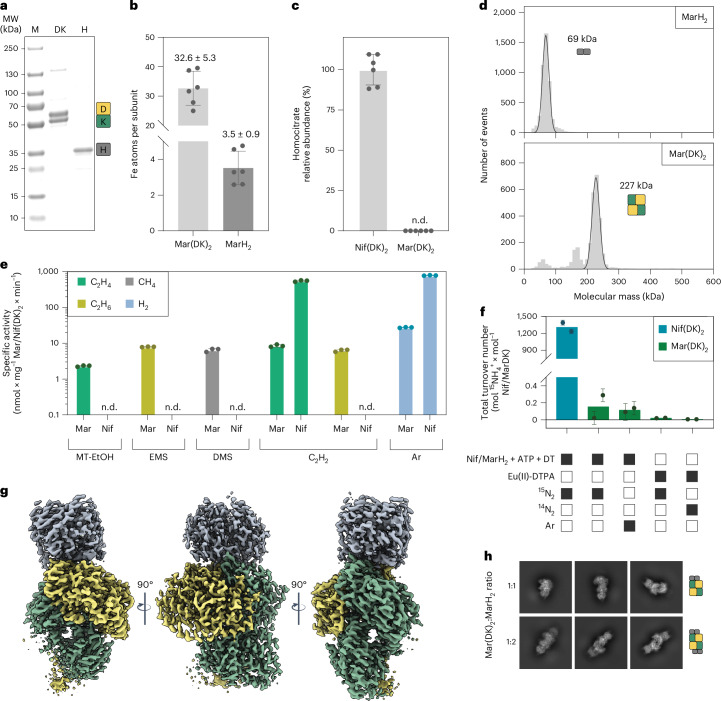
Purification and characterization of the methylthio-alkane reductase. **a**, SDS–PAGE of Strep-tagged MarD (57.5 kDa), MarK (50.8 kDa) and His-tagged MarH (31.9 kDa). MW, molecular weight; M, marker. Purification of the methylthio-alkane reductase complex was reproduced independently with similar results 26 times. **b**, Fe content of Mar(DK)_2_ and MarH_2_ determined by ICP-OES (*n* = 6; independent experiments). **c**, (*R*)-homocitrate abundance in Nif(DK)_2_ and Mar(DK)_2_ determined by HPLC–MS (*n* = 6; independent experiments). **d**, Mass photometry histogram of MarH_2_ (top) and Mar(DK)_2_ (bottom). Event counts are plotted against their corresponding molecular mass. **e**, In vitro activities of the methylthio-alkane reductase (Mar) and Mo-nitrogenase (Nif) for the reduction of MT-EtOH, EMS, DMS, acetylene (C_2_H_2_) and protons under an argon (Ar) atmosphere (*n* = 3; independent experiments). **f**, The methylthio-alkane reductase cannot reduce ^15^N_2_ in vitro. Total turnover numbers for the formation of ^15^NH_4_^+^ by Nif(DK)_2_ or Mar(DK)_2_ depending on reductase component, ATP and sodium DT, or the strong reductant Eu(II)-DTPA detected by NMR; for more details, see Extended Data Fig. 7 (*n* = 2; independent experiments). Columns depict the mean, dots the individual measurements and error bars the propagated instrumental error. **g**, Electron density map of the MarDK_2_H_2_-complex at a global resolution of 2.75 Å (EMD-50553), contoured at level 10. Reductase component is coloured in grey (H_1_ and H_2_), and the catalytic component is coloured in yellow (D_1_) and green (K_1_ and K_2_). **h**, Single-particle cryo-EM 2D classes of the methylthio-alkane reductase with ratios of catalytic:reductase component of 1:1 and 1:2 (for more details, see Extended Data Fig. 3). In **b**, **c** and **e**, columns show the mean, dots the individual measurements and error bars the standard deviation. n.d., not detected.

To reveal the molecular machinery responsible for C**–**S bond reduction of methylthio-alkane reductases (encoded by *marHDK*), we heterologously produced subunits MarH, MarD and MarK from *R. rubrum* and its associated maturation enzyme MarB in an engineered *Rhodobacter capsulatus* strain^17,18^ encoding a disrupted Mo- (*ΔnifD::SpR*) and iron (Fe)-nitrogenases (*ΔanfHDGK::gmR*; for more details, see Methods and Supplementary Table 3). The reductase and catalytic components of the methylthio-alkane reductase were purified separately using affinity chromatography (Fig. 2a). We determined the oligomeric state of each component by mass photometry: the reductase component forms a MarH_2_ homodimer with a measured mass of 69 kDa (Fig. 2d; theoretical mass 64 kDa), while the catalytic component forms a Mar(DK)_2_ heterotetramer with a measured mass of 227 kDa (Fig. 2d; theoretical mass 217 kDa). In conclusion, methylthio-alkane reductase components have the same stoichiometry as the Mo-nitrogenase components.

**Extended Data Fig. 1:**
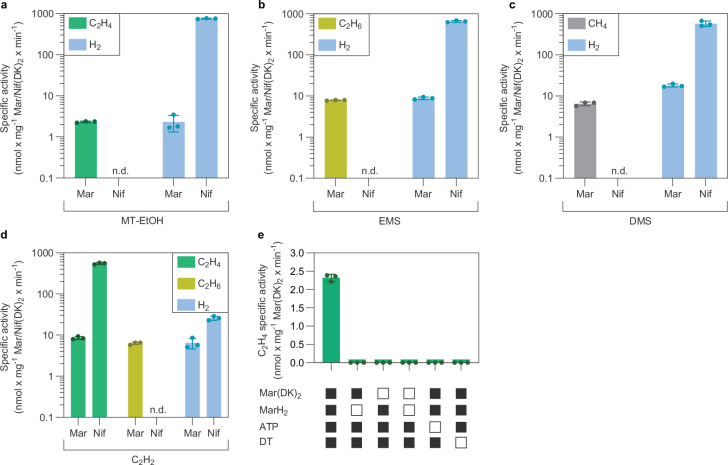
Product formation of methythio-alkane reductase and Mo-nitrogenase. **a–d**, *In vitro* activities of methylthio-alkane reductase (Mar) and Mo-nitrogenase (Nif) for the reduction of (2-methylthio)ethanol (MT-EtOH, **a**), ethyl methyl sulfide (EMS, **b**), dimethyl sulfide (DMS, **c**) and acetylene (C_2_H_2_, **d**). Specific activities are plotted for the formation of ethylene (C_2_H_4_, green), ethane (C_2_H_6_, yellow), methane (CH_4_, gray) and hydrogen (H_2_, blue) (n = 3). n.d., not detected. **e**, *In vitro* C_2_H_4_ specific activity of the methylthio-alkane reductase for the reduction of MT-EtOH is dependent on the addition of reductase and catalytic components, ATP and DT (n = 3). Error bars depict standard deviation of three independent experiments.

The purified enzymes were active for the reduction of MT-EtOH, EMS and DMS as measured by the release of C_2_H_4_, C_2_H_6_ and CH_4_, respectively (Fig. 2e and Extended Data Fig. 1). While the Mo-nitrogenase is unable to reduce VOSCs, the methylthio-alkane reductase converts MT-EtOH at a rate of 2.3 nmol C_2_H_4_ × mg^−1^ Mar(DK)_2 _× min^−1^, EMS at 7.9 nmol C_2_H_6 _× mg^−1^ Mar(DK)_2 _× min^−1^ and DMS at 6.5 nmol CH_4 _× mg^−1^ Mar(DK)_2 _× min^−1^ (Fig. 2e). The methylthio-alkane reductase activity is ATP dependent and requires the addition of both reductase and catalytic components as well as sodium dithionite (DT) as an electron donor (Extended Data Fig. 1e). Intriguingly, we detected the formation of molecular hydrogen (H_2_) for all tested substrates and in the absence of substrate under an argon atmosphere (Fig. 2e and Extended Data Fig. 1a–d).

While Mo-nitrogenase cannot reduce VOSCs, methylthio-alkane reductase can reduce the alternative nitrogenase substrate acetylene (C_2_H_2_) to C_2_H_4_ (Fig. 2e and Extended Data Fig. 1d). Moreover, methylthio-alkane reductase can fully reduce C_2_H_2_ to C_2_H_6_, a characteristic of the alternative nitrogenases, vanadium (V)- and Fe-nitrogenases^19,20^, which contain V or Fe in the position of Mo in FeMoco, termed FeVco and FeFeco^21^. This suggests the presence of FeFeco/FeVco-like cofactors in the methylthio-alkane reductase.

**Fig. 3:**
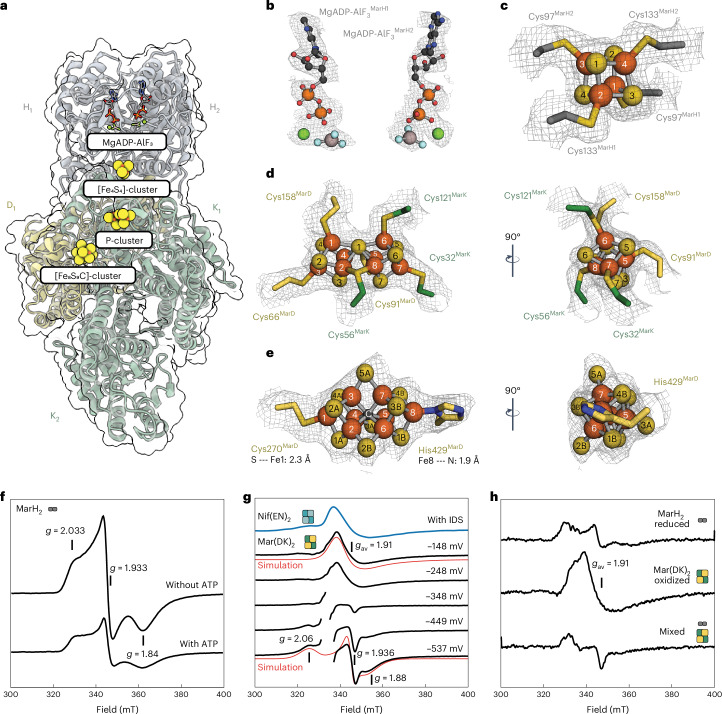
Methylthio-alkane reductase contains nitrogenase cofactors in the active site. **a**, Protein model of the MarDK_2_H_2_-complex with highlighted ligand positions (PDB: 9FMG). Colouring as in Fig. 2g. **b**–**e**, Magnified views of the methylthio-alkane reductase MgADP-AlF_3_ moieties (**b**), the [Fe_4_S_4_]-cluster (**c**), the P-cluster (**d**) and the [Fe_8_S_9_C]-cluster (**e**). Cryo-EM electron density maps are displayed as a grey mesh at contour level 8. Ligands are represented as ball-and-sticks with carbon atoms coloured in dark grey, nitrogen in blue, oxygen in red, phosphorus in orange, aluminium in light grey, fluoride in pale cyan, magnesium in green, sulfur in yellow and iron in dark orange. Amino acid residues coordinating the metalloclusters are shown in stick representation. **f**–**h**, EPR spectroscopy of MarH_2_ and Mar(DK)_2_ metalloclusters: EPR *S* = 1/2 signals of the [Fe_4_S_4_]^1+^ cluster in DT-reduced MarH_2_ with and without MgATP recorded at 10 K (**f**); EPR spectra at 10 K of Mar(DK)_2_ samples poised at the indicated redox potentials show two main species between −550 and −150 mV, and with simulations in red lines (the signal appearing upon oxidation is similar to that of the [Fe_8_S_9_C]-cluster of *A. vinelandii* Nif(EN)_2_ (blue trace)) (**g**); EPR spectra of reduced (DT-free) MarH_2_ (upper trace), oxidized (IDS-free) Mar(DK)_2_ (middle trace) and a mixture of both (lower trace) at 10 K (**h**). Reduced MarH_2_ can transfer electrons to oxidized Mar(DK)_2_ leading to the disappearance of the broad [Fe_8_S_9_C]-cluster-like *g* = 1.91 EPR signal, with concomitant appearance of a sharp rhombic species. EPR conditions: microwave frequency 9.35 GHz; modulation frequency, 100 kHz; 1.5 (**f** and **g**) and 1.0 (**h**) mT modulation amplitude; microwave power 0.2 mW. An isotropic radical was subtracted from all spectra to facilitate simulation and comparison.

**Extended Data Fig. 2:**
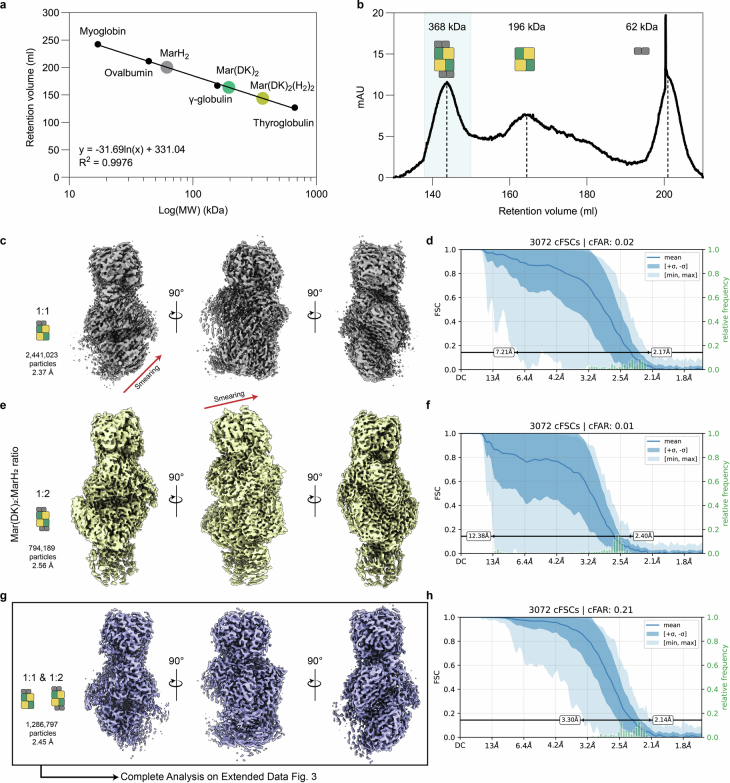
Mar(DK)_2_(H_2_)_2_-complex disassembles into Mar(DK)_2_H_2_-complex with complementary orientations. **a-b**, Isolation of the MgADP-AlF_3_-trapped Mar(DK)_2_(H_2_)_2_-complex by size exclusion chromatography. **a**, Molecular weight (MW, kDa) calibration curve for the size exclusion chromatography column HiLoad 26/600 Superdex 200 pg column (Cytiva, Marlborough, Massachusetts, USA) used to isolate the MgADP-AlF_3_-trapped Mar(DK)_2_(H_2_)_2_-complex for cryo-EM grid preparation. Protein standards are colored in black. Measured complex, reductase and catalytic components are colored in yellow, grey and green, respectively. **b**, Elution profile of the Mar(DK)_2_(H_2_)_2_-complex reaction. The protein fraction used for cryo-EM grid preparation is highlighted in mint. **c**, Electron density maps of the hexameric Mar(DK)_2_H_2_-complex, **e**, the octameric Mar(DK)_2_(H_2_)_2_-complex, and **g**, a particle from combining both populations during cryo-EM analysis. **d**,**f**,**h**, Conical Fourier Shell Correlation (FSC) Area Ratio (cFAR) plots by CryoSPARC v4.4.1 assessing specimen orientation bias for the electron density maps of panels **c**, **e** and **g**, respectively. The line depicts the mean, the dark blue area the standard deviation and the aquamarine area the minimum and maximum value of the correlations at each special frequency. The octameric (**c, d**) and hexameric (**e, f**) complexes separately resulted in electron density maps displaying the smearing effect of preferential orientation in different planes. Combining both populations enables the reconstruction of a particle with an improved electron density map, free from the smearing effect (**g, h**), which was selected for further analysis (see Extended Data Fig. 3).

**Extended Data Fig. 3:**
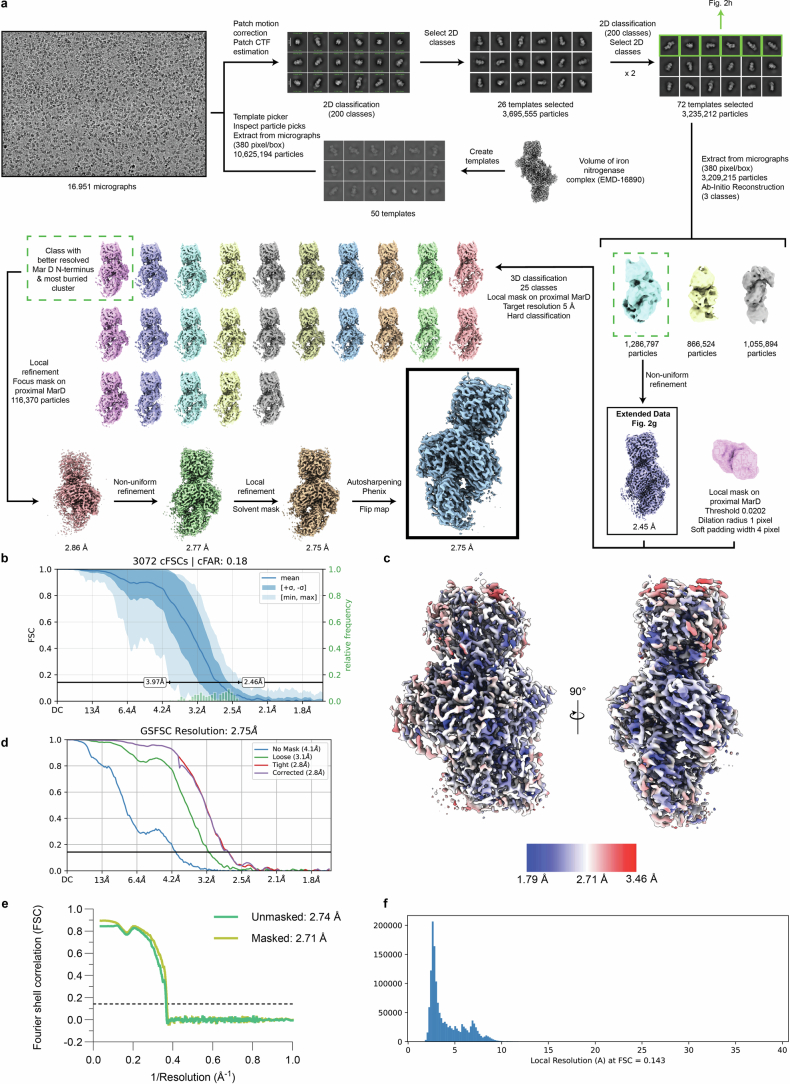
Cryo-EM data acquisition and analysis of the methylthio-alkane reductase complex. **a**, Schematic of the processing workflow for cryo-EM data using CryoSPARC v4.4.1^60^ and Phenix v1.21.1^61^. **b**, Conical FSC Area Ratio (cFAR) plot by CryoSPARC v4.4.1 assessing specimen orientation bias. The line depicts the mean, the dark blue area the standard deviation and the aquamarine area the minimum and maximum value of the correlations at each special frequency. **c**, Local resolution estimated by CryoSPARC v4.4.1 mapped onto the final electron density map contoured at level 10. **d**, Gold-Standard Fourier Shell Correlation (GSFSC) plot by CryoSPARC v4.4.1 with resolution calculated at Fourier shell correlation (FSC) = 0.143. **e**, Map to atomic model FSC plot determined at FSC = 0.143. **f**, Histogram of local resolution values calculated at FSC = 0.143 by CryoSPARC v4.4.1.

To reveal the metalloclusters of Mar(DK)_2_ involved in catalysis, we determined the molecular structure of the methylthio-alkane reductase complex by anaerobic single-particle cryo-EM. We were unable to obtain the structure of the catalytic component alone, due to severe preferential orientation bias and susceptibility to denaturation of MarD through contact with the gas–water interface. We overcame this issue by forming a complex of Mar(DK)_2_ with MarH_2_, using the non-hydrolysable MgATP mimic MgADP-AlF_3_, thus protecting the catalytic component from denaturation. Reference-free two-dimensional (2D) class averages revealed the existence of two major oligomeric states: an octameric Mar(DK)_2_(H_2_)_2_-complex, and a hexameric Mar(DK)_2_H_2_-complex (Fig. 2h). Attempts to resolve either complex in three dimensions were still hampered by severe preferential orientation bias, leading to uninterpretable reconstructions (Extended Data Fig. 2c–f). However, we realized that the preferred orientations of the two oligomeric states were perpendicular to each other. This insight prompted a revised classification strategy, where instead of segregating particles based on oligomeric state, we combined all particles thus resolving structural regions present across both complexes. (Extended Data Fig. 2g,h). This approach led to a substantial improvement in the reconstructed density, but the MarD subunits remained poorly resolved due to particle heterogeneity and the intrinsic flexibility of the N-terminal region. Given our goal to resolve the active site metalloclusters of the methylthio-alkane reductase, we prioritized solving a three-dimensional (3D) class that represented a stable state of one MarD subunit, at the expense of resolving the second copy. By applying strict 3D classification on 25 classes using a local mask on the proximal MarD, we selected the class with the best-resolved N terminus of MarD—specifically, the class in which the active site cluster is most deeply buried within the protein. This enabled a focused refinement of the MarDK_2_H_2_ fragment of the entire Mar complex, yielding a final reconstruction with a global resolution of 2.75 Å. (Fig. 2g and Extended Data Fig. 3). The resulting map enabled us to build a structural model with well-resolved metalloclusters (Fig. 3a), revealing the molecular basis of the methylthio-alkane reductase specificity.

### Methylthio-alkane reductase contains nitrogenase cofactors

**Fig. 4:**
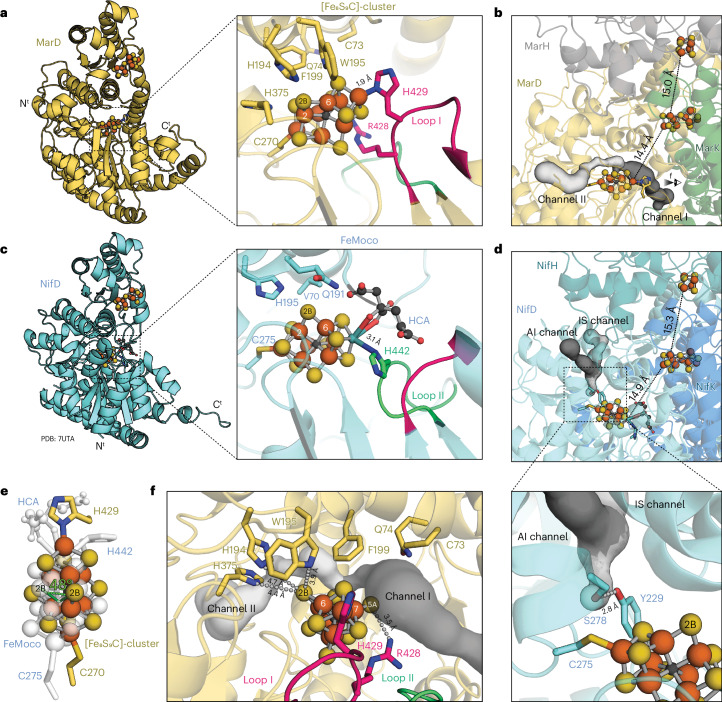
Comparison of active sites from methylthio-alkane reductase and Mo-nitrogenase. **a**,**c**, The active site of the methylthio-alkane reductase (**a**) features an extended loop (Loop I, highlighted in pink) containing His429^MarD^, which coordinates the [Fe_8_S_9_C]-cluster. Conversely, for Mo-nitrogenase (**c**; PDB: 7UTA) a different structural domain (Loop II, highlighted in green) contains His442^*Av*NifD^, which coordinates FeMoco. Both panels display key amino acid residues potentially involved in metallocluster stabilization and catalysis in stick representation. Metalloclusters and (*R*)-homocitrate (HCA) are shown as ball-and-sticks. N^t^, N terminus; C^t^, C terminus. **b**,**d**, Substrate channels predicted by CAVER^36^ accessing the active sites of the methylthio-alkane reductase (**b**) and the Mo-nitrogenase (**d**) are depicted, with the first and second most likely channels coloured in dark and light grey, respectively. Mo-nitrogenase channels correspond to the proposed substrate channels by Igarashi and Seefeldt (IS channel)^33^ and Morrison et al. (AI channel)^34^. Residues that gate the proximal pocket of the Mo-nitrogenase active site are shown in stick representation^35^. Distances between the three metalloclusters, [Fe_4_S_4_]-cluster, P-cluster and active site cofactor, are indicated by dotted lines. **e**, Overlay of [Fe_8_S_9_C]-cluster and FeMoco from the alignment of MarD and NifD, highlighting the displacement of the S2B belt sulfur position between both clusters. **f**, Side view of the active site of the methylthio-alkane reductase emphasizing S2B and S5A as potential substrate recognition and reduction sites. Distances to S2B and S5A from relevant amino acids are shown as a dashed grey line. Colouring as in Fig. 3a.

**Extended Data Fig. 4:**
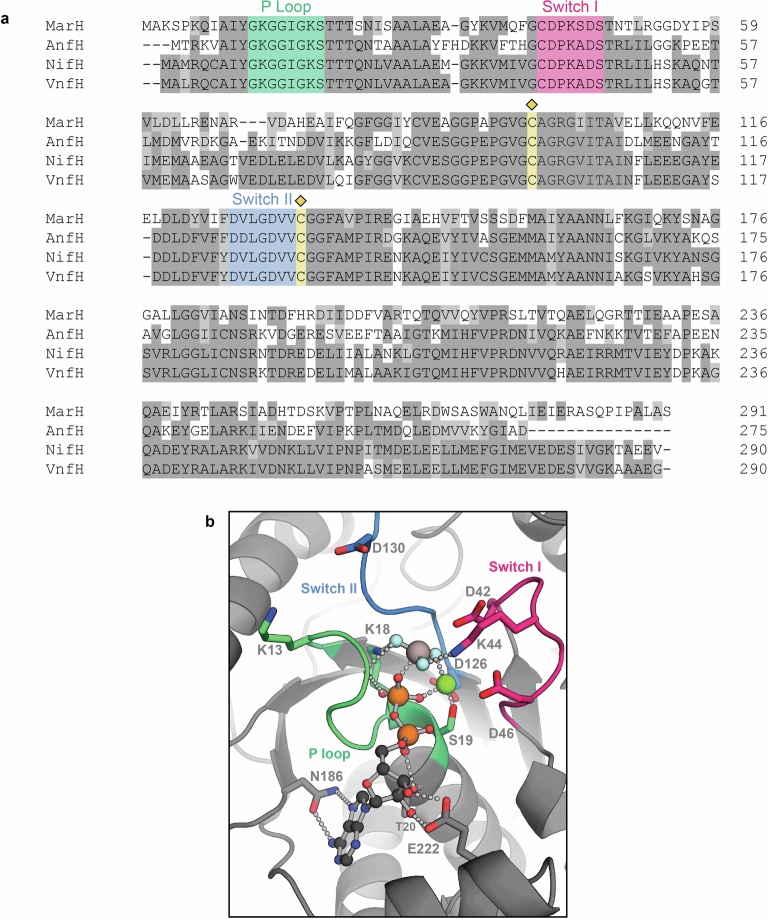
The reductase component MarH_2_ belongs to the family of P-loop NTPases. **a**, Clustal Omega^74,75^ multiple sequence alignment (MSA) of the *R. rubrum* methylthio-alkane reductase reductase component MarH to the *A. vinelandii* Mo-, V- and Fe-nitrogenase reductase components NifH, VnfH and AnfH, colored with Boxshade v3.3. The conserved P-loop, switch I and switch II regions involved in nucleotide binding and hydrolysis are colored in green, pink and blue, respectively. Cys97^MarH^ and Cys133^MarH^ coordinating the [Fe_4_S_4_]-cluster are highlighted in yellow. **b**, Close-up view of the MgADP-AlF_3_ moiety bound to one MarH subunit. P-loop, switch I and switch II regions are colored as in (**a**). MgADP-AlF_3_ is represented in ball-and-stick style with carbon atoms colored in dark gray, nitrogen in blue, oxygen in red, phosphorus in orange, aluminium in light gray, fluoride in pale cyan and magnesium in green. Amino acid residues coordinating the ligand by hydrogen bonding are shown in stick representation.

Analysing the structure, we observed a canonical reductase component MarH_2_, as detailed in Supplementary Discussion 1, containing one MgADP-AlF_3_ moiety per monomer (Fig. 3b and Extended Data Fig. 4) and a single [Fe_4_S_4_]-cluster in its dimeric interface coordinated by Cys97^MarH^ and Cys133^MarH^ (Fig. 3c). Presumably, electrons are transferred from the [Fe_4_S_4_]-cluster to the P-cluster located at a distance of ~15.0 Å, bridging the proximal MarDK dimer (Fig. 3b). The P-cluster is symmetrically coordinated by Cys32^MarK^, Cys56^MarK^, Cys121^MarK^, Cys66^MarD^, Cys91^MarD^ and Cys158^MarD^, featuring a centrally shared sulfide (Fig. 3d) and is most likely present in the reduced state (P^N^), as detailed in Supplementary Discussion 2. Most excitingly, we observed strong electron density at the active site of the methylthio-alkane reductase, located ~14.4 Å away from the P-cluster (Figs. 3e and 4b). The density neatly accommodates the [Fe_8_S_9_C]-cluster from Nif(EN)_2_, the FeMoco precursor, with no density corresponding to any organic moiety. In addition, we were unable to detect (*R*)-homocitrate by high-performance liquid chromatography–mass spectrometry (HPLC–MS) of Mar(DK)_2_ (Fig. 2c). To determine the metal composition of these metalloclusters, we performed inductively coupled plasma optical emission spectroscopy (ICP-OES; Fig. 2b). ICP-OES analysis revealed 32.6 ± 5.3 Fe atoms per catalytic component, consistent with the presence of two P-clusters ([Fe_8_S_7_]-clusters) and two [Fe_8_S_9_C]-clusters per Mar(DK)_2_ heterotetramer, and neither Mo nor V was detected. The reductase component MarH_2_ contained 3.5 ± 0.9 Fe atoms, indicating the presence of a labile [Fe_4_S_4_]-cluster. Thus, methylthio-alkane reductase is the only Nfl enzyme described to harbour the large and complex nitrogenase P- and [Fe_8_S_9_C]-clusters.

### Distinct metallocluster coordination and surrounding

**Extended Data Fig. 5:**
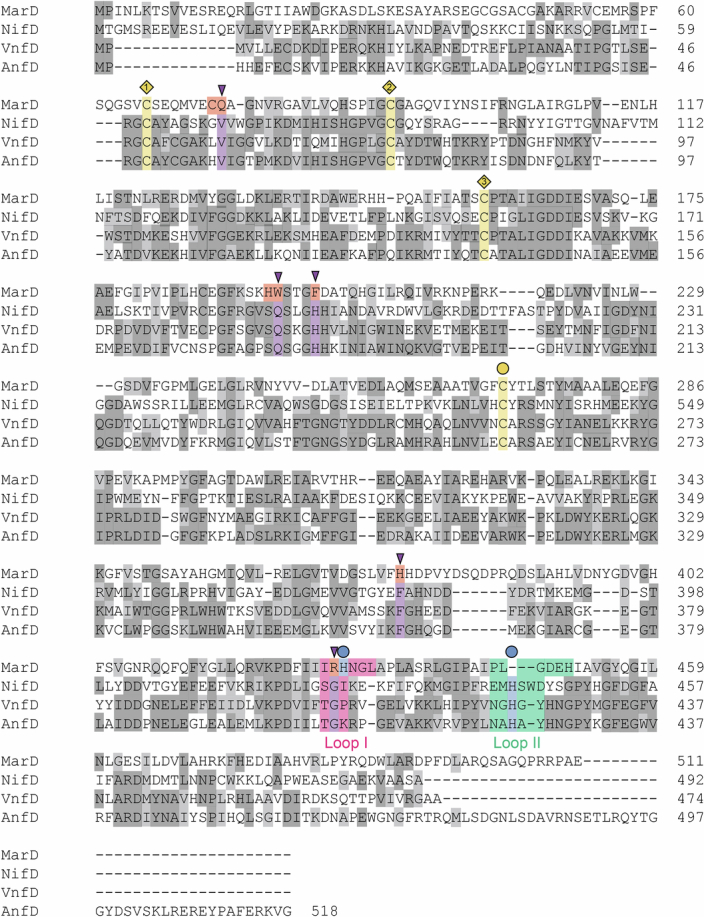
Sequence alignment of D-subunits of methylthio-alkane reductase and nitrogenases. MSA of the *R. rubrum* methylthio-alkane MarD subunit and the *A. vinelandii* Mo-, V- and Fe-nitrogenase NifD, VnfD and AnfD subunits, colored with Boxshade v3.3. Cysteine triads involved in P-cluster coordination are highlighted in yellow and numbered 1-3. Cysteine and histidine ligands responsible for the coordination of the [Fe_8_S_9_C]-cluster, FeMoco, FeVco and FeFeco are labeled with a yellow and blue circle, respectively. Strictly conserved residues involved in nitrogenase catalytic mechanism and FeMoco, FeVco and FeFeco stability are highlighted in purple, while residues replacing them in the structure of the methylthio-alkane reductase are highlighted in orange. The extended loop I of the methylthio-alkane reductase containing the coordinating histidine His429^MarD^ is highlighted in pink, while the Mo-nitrogenase loop II containing the coordinating histidine His422^*Av*NifD^ is highlighted in green.

The core of the [Fe_8_S_9_C]-cluster consists of a trigonal prism formed by Fe2–Fe7, which are bridged by three belt sulfides. Due to the presence of all conserved NifB motifs in MarB and the presence the radical SAM domain responsible for carbide insertion^4^, we assume a similar role of MarB and NifB in the assembly of the nitrogenase precursor cluster^22^. Thus, an interstitial carbide was placed in the centre of the cluster, although the resolution of the electron density map is insufficient to locate the carbide. The metallocluster is anchored to the protein scaffold by two amino acid residues, Cys270^MarD^ to Fe1 and His429^MarD^ to Fe8 (Fig. 4a), analogous to the coordination of Fe1 and Mo of FeMoco by Cys275^*Av*NifD^ and His442^*Av*NifD^, respectively^23^ (Fig. 4c). However, His429^MarD^ is located at an extended loop specific to MarD, which is not conserved in bona fide nitrogenases (Loop I in Fig. 4a,f and Extended Data Fig. 5). This explains why previous sequence analysis of the methylthio-alkane reductase gene cluster had failed to identify this ligand^4^. The octahedral coordination of Mo in the Mo-nitrogenase, which involves three sulfides, (*R*)-homocitrate and His442^*Av*NifD^, is replaced in the [Fe_8_S_9_C]-cluster of the methylthio-alkane reductase by a tetrahedral coordination due to the absence of (*R*)-homocitrate. As a result, the anchoring to the protein adopts an axial geometry, and the bond distance between His429^MarD^ and Fe8 of the [Fe_8_S_9_C]-cluster is shorter (1.9 Å) compared with the bond distance between His442^*Av*NifD^ and the Mo atom of FeMoco (3.1 Å in PDB: 7UTA (ref. ^23^)). This is also the case when compared with the bond distance between His423^*Rc*AnfD^ and Fe8 of FeFeco from the Fe-nitrogenase (2.7 Å in PDB: 8OIE (ref. ^17^)). The observed coordination of the methylthio-alkane reductase [Fe_8_S_9_C]-cluster by just two residues (Cys270^MarD^ and His429^MarD^) indicates that this cluster indeed necessitates a carbide in its centre, because the geometry of an [Fe_8_S_9_]-cluster with six trigonal Fe atoms would not be possible without the structural support of a shared central ligand.

**Extended Data Fig. 6:**
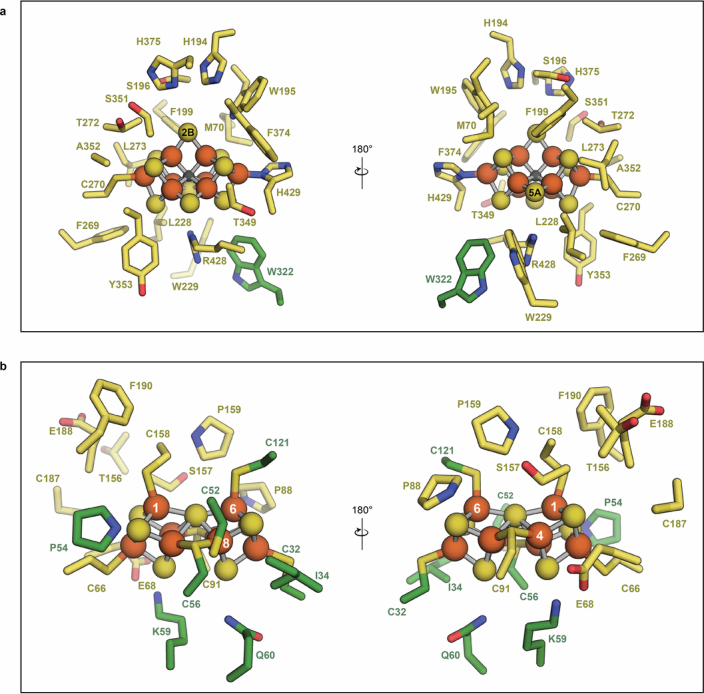
Environment of the methylthio-alkane reductase [Fe_8_S_9_C]-cluster and P-cluster. **a,b**, Close-up view of the [Fe_8_S_9_C]-cluster of the active site of the methylthio-alkane reductase (**a**) and the P-cluster bridging the MarDK subunits (**b**). Metalloclusters are shown as ball-and-sticks, with carbon atoms in dark gray, sulfur in yellow, and iron in dark orange. Amino acid residues surrounding the metalloclusters within a 5 Å radius are shown in stick representation. Amino acid residues of MarD and MarK are colored in yellow and green, respectively.

Beyond the distinct cluster composition, the direct protein environment of methylthio-alkane reductases also deviates from nitrogenases. Substrate binding to nitrogenase metalloclusters has been proposed to occur between Fe2 and Fe6, where the bridging sulfide S2B is replaced by the substrate^24–28^. The conserved residues Val70^*Av*NifD^, Gln191^*Av*NifD^ and His195^*Av*NifD^ surround S2B and are essential for N_2_ reduction^29^ (Fig. 4c). The cavity left by the small side chain of Val70^*Av*NifD^ allows substrate access to Fe6 of FeMoco^30^. His195^*Av*NifD^ forms a hydrogen bond with S2B in the resting state and possibly donates protons during catalysis, while Gln191^*Av*NifD^ undergoes structural rearrangements that allow S2B displacement by the substrate during turnover^26,31^. The residues lining the active site of the methylthio-alkane reductase differ substantially (Extended Data Fig. 6a): Cys73^MarD^ and Gln74^MarD^ replace Val70^*Av*NifD^, while tryptophan (Trp195^MarD^) and phenylalanine (Phe199^MarD^) residues substitute Gln191^*Av*NifD^ and His195^*Av*NifD^, respectively (Fig. 4a). However, because the S2B site of the [Fe_8_S_9_C]-cluster of the methylthio-alkane reductase is rotated by ~48° compared with the S2B site of FeMoco taking as the vertex its interstitial carbide, only Trp195^MarD^ is close enough to S2B to form a hydrogen bond (Fig. 4e,f). Trp195^MarD^, His194^MarD^ and His375^MarD^ surround the S2B site of the [Fe_8_S_9_C]-cluster at distances of 3.9–4.7 Å and thus could be involved in substrate recognition and binding as well as proton transfer during turnover (Fig. 4a,f). The [Fe_8_S_9_C]-cluster in Mar(DK)_2_ is also stabilized by Arg428^MarD^, which forms a hydrogen bond with the belt sulfur S5A (Fig. 4f). Interestingly, Arg428^MarD^ is located at the extended Loop I of MarD, adjacent to the [Fe_8_S_9_C]-cluster coordinating His429^MarD^, and replacing Gly424^*Av*NifD^, a residue strictly conserved in all nitrogenases^32^ (Extended Data Fig. 5). Consequently, Arg428^MarD^ together with His429^MarD^ might be a hallmark of methylthio-alkane reductases and distinguish them from nitrogenases. In summary, the methylthio-alkane reductase active site suggests a different substrate recognition and proton transfer to nitrogenases.

### Substrate channels determine specificity

Differences between methylthio-alkane reductase and Mo-nitrogenase extend to substrate channels: Mo-nitrogenase substrate channels proposed by Igarashi and Seefeldt (IS)^33^ and Morrison et al. (AI)^34^ are restricted from accessing the proximal pocket of FeMoco due to hydrogen bond gating by Tyr229^*Av*NifD^ and Ser278^*Av*NifD^^35^ (Fig. 4d). By contrast, the two most likely methylthio-alkane reductase substrate channels predicted by CAVER^36^ do not show steric impediments. Restricted access to the active site of Mo-nitrogenase could explain its inability to utilize VOSCs, despite being more efficient at reducing smaller substrates (Fig. 2e). The most likely substrate channel in the methylthio-alkane reductase (channel I) originates at the interface of MarD and MarK, passes the belt sulfide S5A on the way to S2B and occupies the void left by the absent (*R*)-homocitrate (Fig. 4b,f). The second channel (channel II) originates at the surface of MarD opposite to channel I and ends at S2B of the [Fe_8_S_9_C]-cluster (Fig. 4b,f). Nevertheless, given the symmetric geometry of the [Fe_8_S_9_C]-cluster, substrate binding to any of the three belt sulfurs, S2B, S5A and S3A, cannot be discarded at this point. Further investigations are needed to decipher the binding of methylthio-alkanes to the [Fe_8_S_9_C]-cluster during catalysis.

### The [Fe_8_S_9_C]-cluster does not reduce N_2_

**Extended Data Fig. 7:**
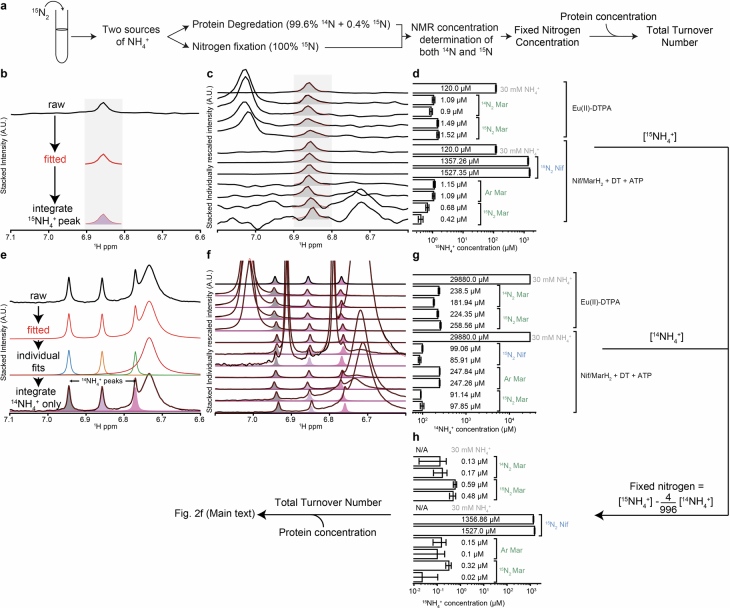
Nitrogen assays with methylthio-alkane reductase and product detection by NMR spectroscopy. Nitrogenases provide two sources of NH_4_^+^: First, by protein degradation, second, by nitrogen fixation. When ^15^N_2_ is used as the nitrogen source, the nitrogen fixation will be isotopically labelled ^15^NH_4_^+^, while protein degradation will remain at natural abundance (99.6% ^14^NH_4_^+^, 0.4% ^15^NH_4_^+^) (**a**). We measured the concentration of both ^14^NH_4_^+^ and ^15^NH_4_^+^ and used natural abundance ratios to distinguish nitrogen fixation from protein degradation, providing a more precise quantification of Total Turnover than simply measuring ^15^NH_4_^+^ (**a**). A single Lorentzian function is fitted (**b**, red) to each processed NMR spectrum from the ^15^N HSQC experiment (**b**, black), before being integrated (**b**, pink shaded) and scaled against reference NH_4_^+^ samples to provide a concentration. Performing this on all samples (**c**) shows good agreement between fitted (**c**, red) and raw (**c**, black) data. The extracted ^15^NH_4_^+^ concentrations span the nanomolar to millimolar range (**d**). n Lorentzian functions are fitted (**e**, red and individual fits) to the processed water-suppressed ^1^H NMR data and the ^14^NH_4_^+^ peaks are extracted by identifying the fitted peak locations split by the characteristic 52.5 Hz coupling constant (**e**, shaded). The summed integral of the triplet is then scaled against reference NH_4_^+^ samples to provide a concentration. This was performed on all samples and the resulting fits (**f**, red) show good agreement with raw (**f**, black) data. The ^14^NH_4_^+^ concentrations span the 100 µM to 250 µM range for the protein samples (**g**). As the protein is isotopically non-enriched, we assume the degradation products will have the natural abundance distribution of 996:4. To account for this, we subtract 0.4% of the ^14^NH_4_^+^ concentration from the ^15^NH_4_^+^ concentration to produce a subtracted ^15^NH_4_^+^ concentration that describes the maximum ^15^NH_4_^+^ concentration originating from nitrogen fixation (**h**). The data was used to calculate total turnover numbers for Fig. 2f. The error bars in **d, g, h** show the propagated instrumental error.

Based on our finding that the methylthio-alkane reductase contains an [Fe_8_S_9_C]-cluster in conjunction with the recent observations that [Fe_8_S_9_C]-cluster-containing nitrogenase maturase Nif(EN)_2_ can reduce N_2_ to NH_3_ to a slight extent^37^, we aimed to determine the N_2_ reduction activity of methylthio-alkane reductases. No formation of ^15^NH_4_^+^ beyond the background could be detected by nuclear magnetic resonance (NMR) in ^15^N_2_-assays mimicking physiological conditions containing catalytic component Mar(DK)_2_, reductase component MarH_2_, ATP and DT, in contrast to Mo-nitrogenase assays under the same conditions (Fig. 2f and Extended Data Fig. 7). Assays with just the catalytic component Mar(DK)_2_ and the strong reductant europium-II-diethylenetriamine pentaacetic acid (Eu(II)-DTPA) also did not display ^15^NH_4_^+^ formation above background levels (Fig. 2f and Extended Data Fig. 7). This result contrasts with the observation that Nif(EN)_2_ can reduce one to three molecules of N_2_ in a reductase component- and Eu(II)-DTPA-dependent manner^37^. Thus, our results reveal that the presence of an [Fe_8_S_9_C]-cluster is not the sole determinant for N_2_ reduction; rather, the coordination and the immediate surroundings of the [Fe_8_S_9_C]-cluster may determine its activity.

### Spectroscopy and redox states of metalloclusters

**Extended Data Fig. 8:**
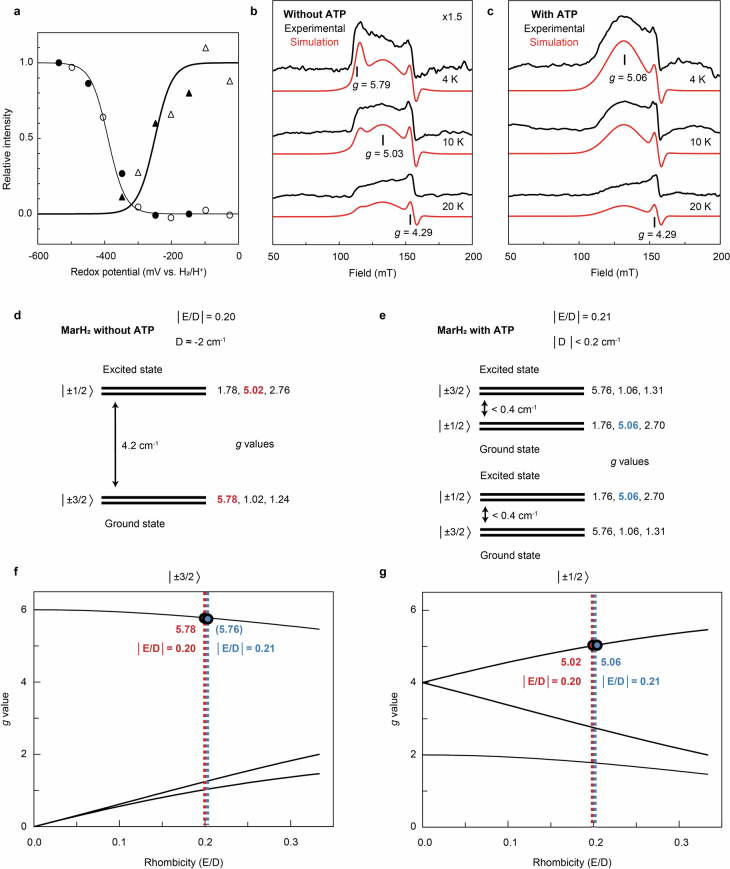
EPR spectroscopy of Mar(DK)_2_ and MarH_2_ metalloclusters. **a**, Amplitudes of the two species from Fig. 3g and further redox titrations of Mar(DK)_2_ with fits to the Nernst equation for n = 1 with E_m_ = −390 and −250 mV. **b,c**, *S* = 3/2 EPR signals of the [Fe_4_S_4_]^1+^ cluster in dithionite (DT)-reduced MarH_2_ with (**c**) and without (**b**) MgATP. EPR signals recorded at 4, 10 and 20 K in black lines, and with simulations in red lines (parameters in Supplementary Table 2). EPR conditions: microwave frequency 9.35 GHz; modulation frequency, 100 kHz; 1.5 mT modulation amplitude; microwave power, 20 mW. **d, e**, Energy level diagrams of the *S* = 3/2 spin system of MarH_2_. The calculated *g*-values for MarH_2_ without ATP (**d**) are in red, those for MarH_2_ in the presence of ATP in blue (**e**). The intensity of the *g* = 5.06 peak shows Curie law behaviour, indicating that both doublets are almost equally populated. Thus, it could not be determined whether the peak is from the ground or excited state, and the magnitude and sign of the D value could not be estimated. The two possible energy level diagrams are shown. **f, g**, Rhombograms for the two |±3/2〉 and |±1/2〉 doublets of the *S* = 3/2 state^76^. Calculated g-values as function of the rhombicity (E/D) are shown.

**Extended Data Fig. 9:**
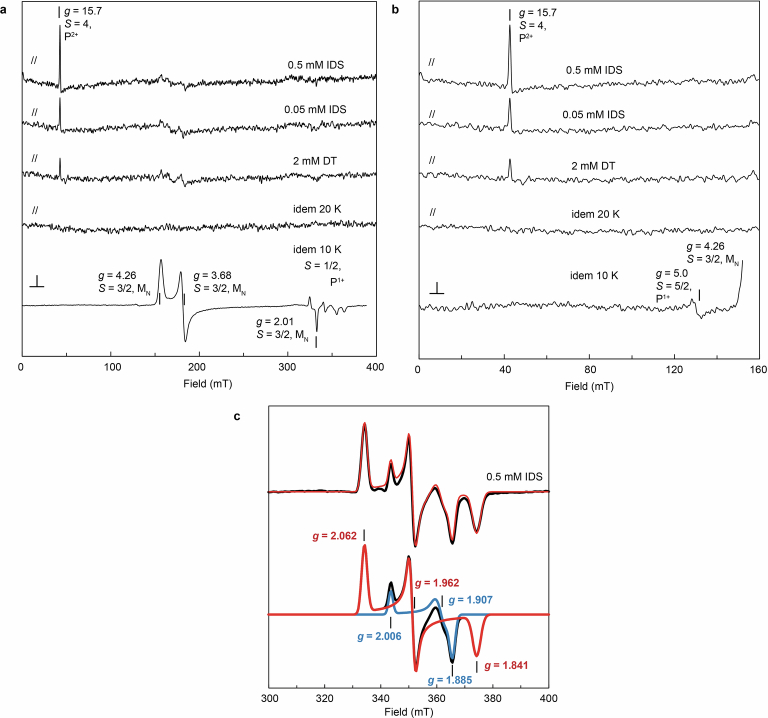
EPR spectroscopy of the metalloclusters of *Rhodobacter capsulatus* Nif(DK)_2_. **a**, Samples of desalted Nif(DK)_2_ were treated with indicated oxidants (5,5′-indigodisulfonate, IDS) or reductant (dithionite, DT) and measured at 4 K (or as indicated otherwise). The strong *S* = 3/2 EPR signal in DT-reduced Nif(DK)_2_ (bottom trace, x0.1 in comparison to the other traces) was accompanied by a weak *S* = 1/2 EPR signal in the measurement with the perpendicular mode of a dual mode cavity. These signals disappear in the parallel mode. However, at 4 K a very sharp *g* = 15.6 signal of the *S* = 4 state of the P^2+^ cluster was detected, which increased several fold upon progressive IDS oxidation. The signal is from an excited state, as its intensity decreased with increased temperature (see trace at 20 K). **b**, Zoom in of (**a**), with the bottom trace on the same scale as the other traces to reveal a weak derivative-shaped *g* = 5.0 signal belonging to the middle g-value of the |±1/2〉 doublet in the *S* = 5/2 state of the P^1+^ cluster. The corresponding broad *g* = 6.7 peak could not be detected. **c**, The *S* = 1/2 EPR signal at 20 K of the P^1+^ cluster in 0.5 mM IDS-oxidized Nif(DK)_2_, which could be simulated by a mixture of two species. Top traces: experimental spectrum (black line) with the sum of the simulated spectra (red line, parameters in Supplementary Table 3). Bottom traces: individual simulations in red and blue lines, together with their sum (black line). EPR conditions: microwave frequency, 9.63 GHz (perpendicular mode) or 9.34 GHz (parallel mode); modulation frequency, 100 kHz; 0.5 mT modulation amplitude, microwave power, 20 mW.

Nitrogenases and Nfl enzymes exhibit very distinct EPR spectroscopic features^13^, which define the redox state and electronic structure of the metalloclusters. At 4–20 K, DT-reduced MarH_2_ exhibited the typical physical spin mixture of a *S* = 1/2 (*g* = 2.03, 1.933 and 1.84) and a *S* = 3/2 species of the [Fe_4_S_4_]^1+^ cluster, characteristic for NifH_2_ (ref. ^38^), of which the relative content of the two spin states is ATP dependent, as detailed in Supplementary Discussion 3 (Fig. 3f and Extended Data Fig. 8b,c). Next, we analysed the catalytic component Mar(DK)_2_: upon DT reduction, and in a dye-mediated redox titration with a midpoint potential (*E*_m_) of −390 mV versus normal hydrogen electrode, a relatively sharp *S* = 1/2 signal (*g*_average_ = 1.96) appeared (Fig. 3g and Extended Data Fig. 8a). Although the Mar(DK)_2_ properties did not match those of the Mo-nitrogenase Nif(DK)_2_ (Supplementary Discussion 4, Extended Data Fig. 9 and Supplementary Table 3), it was very similar to the signal assigned to a reduced P-cluster in the catalytic component of V-nitrogenase^39^. Because the fully intact DT-reduced P^N^ cluster is diamagnetic and the fully intact P^2+^ oxidized state has a *g* = 12 integer spin signal in Nif(DK)_2_ (ref. ^40^) and Vnf(HDK)_2_ (ref. ^41^), this signal probably derives from a subclass of incompletely matured P-clusters containing an [Fe_4_S_4_]^1+^ half^41,42^. Upon oxidation with 5,5′-indigodisulfonate (IDS) or by dye-mediated redox titration with *E*_m_ = −250 mV versus normal hydrogen electrode, a peculiar broad *S* = 1/2 signal (simulated with *g*_*xyz*_ = 1.967, 1.926 and 1.83) with *g*_average_ = 1.91 appeared, totally unlike P^1+^ and P^3+^ EPR signals^13,40^. This EPR signal has previously been observed for the [Fe_8_S_9_C]-cluster-containing nitrogenase maturase Nif(EN)_2_ (ref. ^37^) (Fig. 3g). Our findings show that the *g*_average_ = 1.91 signal is the fingerprint for the active site metallocluster of Mar(DK)_2_ and indicate that a carbide is indeed present in the [Fe_8_S_9_C]-cluster of the methylthio-alkane reductase. By mixing reduced (but DT-free) MarH_2_ with oxidized (but IDS-free) Mar(DK)_2_ and MgATP, we could demonstrate that the structurally defined electron transfer pathway (Fig. 3a) guides an electron to the species with *g*_average_ = 1.91 (Fig. 3h). Concomitantly, the signal assigned to the reduced P-cluster partially appeared, in agreement with its low redox potential.

### Evolution of nitrogenase metalloclusters

**Extended Data Fig. 10:**
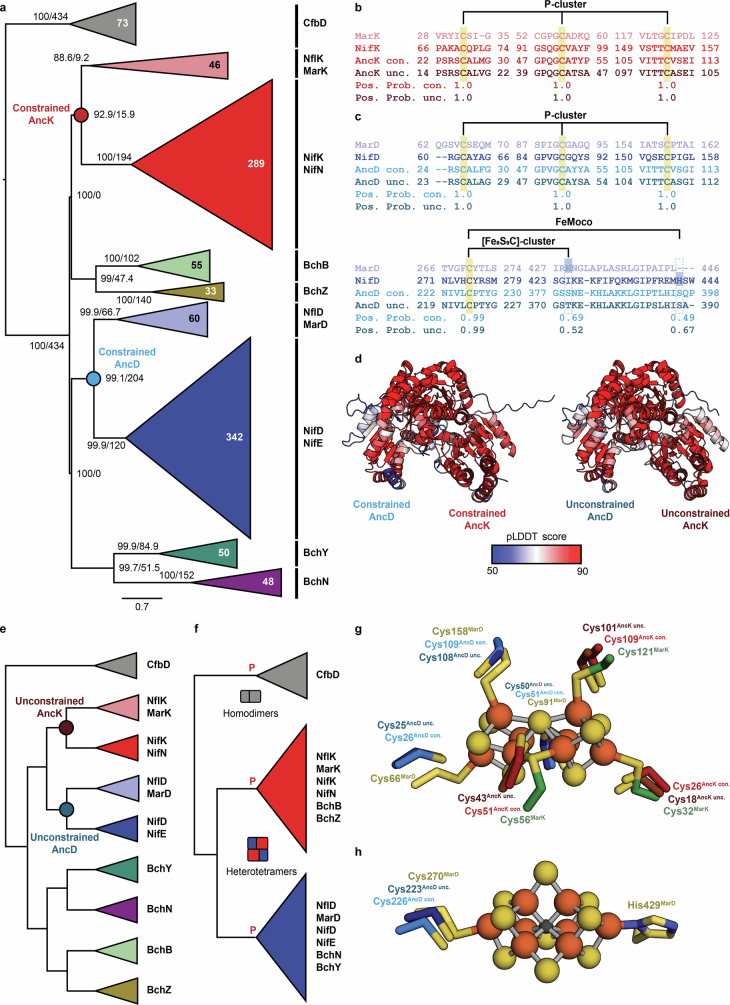
Phylogenetic relationship between nitrogenase and the nitrogen fixation-like protein families. **a**, Maximum likelihood tree of Ni^2+^-sirohydrochlorin *a*,*c*-diamide reductive cyclase complex component D (CfbD), dark-operative protochlorophyllide *a* oxidoreductase (BchNB), chlorophyllide *a* oxidoreductase (BchYZ), nitrogenase-like proteins including the methylthio-alkane reductase (NflDK and MarDK) and Mo-dependent nitrogenase (NifDKEN) subunits. The tree was rooted with homomeric archaeal CfbD and constrained to have BchYN sister to NifDE, NflD, and MarD and to have BchBZ sister to NifKN, NflK and MarK. The mirroring topology of nitrogenases and nitrogenase-like proteins implies an ancestral gene duplication giving rise to ancestral nitrogenase-like proteins and a second, more recent duplication for the emergence of *bona fide* Mo-nitrogenases (NifDKEN). The ancestral nodes resurrected correspond to the last common ancestor of MarD and NifD and the last common ancestor of NifK and MarK. The number of sequences per clade is indicated. Supporting values represent the ultrafast bootstraps replicates by Iqtree2^68^ and the approximate likelihood-ratio (aLRT)^69^ statistical test, respectively. Scale bar represents the number of substitutions per site. **b,c**, Multiple sequence alignments (MSA) of the *Rhodospirillum rubrum* methylthio-alkane reductase MarK subunit to the *A. vinelandii* Mo-nitrogenase NifK subunit and the resurrected ancestral node sequence AncK, from both constrained and unconstrained trees (**b**) and *R. rubrum* MarD, *A. vinelandii* NifD and resurrected ancestral node sequence AncD, from both constrained and unconstrained trees (**c**), showing the binding sites for P-cluster, FeMoco and [Fe_8_S_9_C]-cluster. Pos. Prob., posterior probability. **d**, Structure of the AncDK dimer, from both constrained and unconstrained trees, predicted by AlphaFold2^70^ and colored by predicted local distance difference (pLDDT) score. **e**, Simplified cladogram of the unconstrained tree topology. **f**, Schematic representation of the applied constrain, P indicates where polytomies were introduced. This topology was chosen for analysis due to the more parsimonious history in terms of gene duplication: all nitrogenase relatives other than CfbD are heterotetramers made from two paralogs. **g, h**, Close-up of the methylthio-alkane reductase P-cluster (**g**) and [Fe_8_S_9_C]-cluster (**h**) aligned to the predicted structure of the ancestor AncDK dimer. Amino acid residues coordinating the metalloclusters are shown in stick representation and colored in yellow, green, blue and red for MarD, MarK, AncD and AncK, respectively. An uncollapsed phylogeny with sequence identifiers, a separate file with identifiers, as well as the multiple sequence alignment are available as source data files.

Our structural and biophysical data conclusively show that methylthio-alkane reductases contain metalloclusters until now exclusively associated with nitrogenases. This strongly suggests that their last common ancestor probably contained a P-cluster and a large active site cluster, as previously hypothesized^43^. The fact that we find ligating cysteines in methylthio-alkane reductases that are in homologous positions to equivalent cysteines in nitrogenases strongly suggests that these ligation sites already existed in their last common ancestor. To test this prediction, we first inferred a phylogeny of the subunits assembling the catalytic component of the wider nitrogenase family, composed of nitrogenase-like proteins (NflDK) including the methylthio-alkane reductase (MarDK), Mo-nitrogenase (NifDK), FeMoco maturase (NifEN), dark-operative protochlorophyllide *a* oxidoreductase (BchNB), chlorophyllide *a* oxidoreductase (BchYZ) and Ni^2+^-sirohydrochlorin *a*,*c*-diamide reductase (CfbD). Nitrogenases and their relatives are heterotetrameric proteins comprising two paralogues—usually called D and K. This arrangement is a result of an ancient gene duplication event of an ancestrally likely homomeric protein, represented today by CfbD_2_ (ref. ^43^). Subsequent operon duplications then produced the different paralogous heterotetramers known in this family. CfbD is an outgroup to all other sequences, which roots the tree between the K and D paralogues of the catalytic components of methylthio-alkane reductase and nitrogenase. BchNB and CfbD (just under 30% of all sequences from our alignment) neither have all cysteines conserved nor contain a P- or [Fe_8_S_9_C]-cluster. Our initial tree search yielded a maximum-likelihood tree in which all Bch proteins are a sister group to all nitrogenase(-like) proteins including methylthio-alkane reductases (Extended Data Fig. 10e). This implies that the duplication from homomers to paralogous heteromers occurred twice, once in nitrogenase(-like) proteins and once in Bch proteins. The support for this unparsimonious split was low, so we inferred another tree in which we constrained the topology such that Bch and nitrogenase(-like) proteins including methylthio-alkane reductases underwent only one initial duplication from a homomer, followed by subsequent operon duplications (Extended Data Fig. 10a). This constrained tree was not rejected by the approximately unbiased test, and we view it as more plausible. We then used ancestral sequence reconstruction to infer sequences of the two subunits (D and K) that together assembled into the last common ancestor of nitrogenases and methylthio-alkane reductases for the unconstrained and constrained trees. Our reconstructions of these two very deep nodes were not confident enough across their entire sequence to resurrect this ancient protein experimentally (average posterior probability across sites 0.70). Posterior probabilities were much higher, however, around the crucial metallocluster binding sites. We confidently infer the six cysteines that would ligate a P-cluster with a posterior probability of 1 for both trees, which are in positions homologous to their positions in methylthio-alkane reductases and nitrogenases. For the residues ligating the [Fe_8_S_9_C]-cluster, we confidently infer a cysteine homologous to Cys270^MarD^ (posterior probability of 0.99), but not a histidine equivalent to His429^MarD^ or His442^*Av*NifD^, because this ligand is located in different loops in nitrogenases and methylthio-alkane reductases (Extended Data Fig. 10). Our results indicate that this common ancestor had a P-cluster and probably an [Fe_8_S_9_C]-cluster ligated differently than extant nitrogenases and methylthio-alkane reductases. This ancestral reductase was probably catalytically active and contained a complex metallocluster buried in its active site, rather than being a maturase with a surface-exposed cluster like Nif(EN)_2_ (ref. ^15^), although its activity is unresolved, so far. The discovery of nitrogenase clusters in the methylthio-alkane reductase provides a new perspective on how the protein scaffold controls the maturation, insertion and reactivity of these great clusters of biology^44^ and strongly suggests that their history predates the origin of biological N_2_ fixation by bona fide nitrogenases as we know them today. This is consistent with the recent discovery that [Fe_8_S_9_C]-clusters are part of the activation complex of methyl-coenzyme M reductase, which probably preceded the evolution of [Fe_8_S_9_C]-cluster-bearing nitrogenase(-like) enzymes^45^.

## Discussion

We aimed to identify the factors determining the unique activities of methylthio-alkane reductases. We revealed that these enzymes contain large nitrogenase metalloclusters, including a P-cluster bridging the MarDK subunits and an [Fe_8_S_9_C]-cluster in the active site of MarD, instead of an [Fe_4_S_4_]-cluster and a substrate-binding site. Consequently, methylthio-alkane reductase is the only Nfl enzyme with such metalloclusters that cannot perform N_2_ fixation. The absence in methylthio-alkane reductase of conserved nitrogenase amino acid residues Val70^*Av*NifD^, Gln191^*Av*NifD^ and His195^*Av*NifD^ involved in the N_2_-reduction mechanism^29^ could explain this observation. Moreover, complete nitrogenase activity might require (*R*)-homocitrate, suggested to be crucial for proton transfer during Mo-nitrogenase catalysis^46–48^. While Mo-nitrogenase is probably more reactive and efficient in electron transport, it cannot break the weaker C–S bond of VOSCs reduced by methylthio-alkane reductases. Our structural data suggest that the specificity of methylthio-alkane reductases for methylated sulfur compounds is probably due to wider substrate channels in comparison with nitrogenases, allowing unrestricted access of these larger compounds to the active site. The insights gained in this study highlight the catalytic potential of the [Fe_8_S_9_C]-cluster beyond ammonia formation and open the door to engineering the methylthio-alkane reductase for the biotechnological production of small hydrocarbons such as methane and ethylene.

## Methods

### Chemicals

Chemicals were acquired from Thermo Fisher Scientific, Carl Roth, Sigma-Aldrich and Honeywell. Gas bottles were purchased from Air Liquide.

### Bacterial strains and growth conditions

Modified *Rhodobacter capsulatus* B10S^49^ strain MM0246^17^ (*ΔnifD::SpR ΔanfHDGK::gmR ΔdraTG ΔmodABC ΔgtaI*) was used for the heterologous expression and purification of the methylthio-alkane reductase from *R. rubrum*. *R. capsulatus* MM0246 does not encode for a methylthio-alkane reductase^4^ and was designed for the optimal plasmid-based production of nitrogenase-like enzymes under the Fe-nitrogenase *anfH* promoter. The advantageous genetic modifications of this production strain include the disruption of native Mo-nitrogenase *nifD* and Fe-nitrogenase *anfHDGK* genes with a spectinomycin or a gentamycin resistance cassete, respectively^17^, the deletion of the nitrogenase posttranslational modification machinery *draTG* responsible for the reversible inhibition of the nitrogenase reductase component by ADP ribosylation^50^, the deletion of the *modABC* transporter responsible for the cellular uptake of Mo, a strong repressor of the *anfH* promoter^51^, and the deletion of the quorum sensing gene *gtaI* responsible for the formation of the capsule, which improves cell pelleting of *R. capsulatus* during collection^52^. *R. capsulatus* was cultured anaerobically under photoheterotrophic conditions (60 W krypton lamps) in modified *R. capsulatus* Minimal Medium V (RCV) liquid medium^17^ with the addition of 10 mM serine and 20 µg ml^−1^ streptomycin sulfate.

*Escherichia coli* DH5α (Thermo Fisher Scientific) and *E. coli* ST18 (ref. ^53^) were used for expression plasmid cloning and conjugation into *R. capsulatus* by diparental mating^54^. A list of all strains used in this study is found in Supplementary Table 4.

### Molecular cloning

The *marBHDK* gene cluster (Rru_A0793–Rru_A0796) was amplified from *R. rubrum* DNA by PCR with Q5 high-fidelity DNA polymerase (New England Biolabs) and cloned under the *R. capsulatus anfH* promoter via Golden Gate assembly^55^ using NEBridge Golden Gate Assembly Kit (New England Biolabs) into a broad-host range plasmid pOGG024 (ref. ^56^) (pMM0165). To purify the methylthio-alkane reductase subunits separately, affinity tags were added to the sequences via Gibson Assembly^57^ using NEBuilder HiFi DNA Assembly Master Mix (New England Biolabs; pMM0181). A hexahistidine tag was added to the N terminus of MarH and a Strep-tag II to the C terminus of MarD, to purify reductase and catalytic components^58^. The sequence of pMM0165 and pMM0181 were confirmed by whole-plasmid sequencing (Plasmidsaurus). Primers and plasmids used in this study are listed in Supplementary Tables 5 and 6.

### Protein purification and characterization

Purification of Strep-tagged Mar(DK)_2_ and His-tagged MarH_2_ from *R. capsulatus* MM0422 was started by inoculating at an optical density at 660 nm (OD_660_) of 0.05 modified RCV medium^17^ with 10 mM serine and 50 µg ml^−1^ kanamycin sulfate. Liquid cultures were cultivated anaerobically under illumination in 1.2 atm of 100 % argon atmosphere at 30 °C until reaching a final OD_660_ of ~3.2. Cultures were collected inside an anaerobic COY tent (COY Laboratory Products), with a >95% Ar <5% H_2_ atmosphere and were spiked with 2 mM sodium DT. Cell pelleting was performed in a Beckman Coulter centrifuge (Beckman Coulter) at 10,000*g*, for 30 min at 10 °C. The supernatant was discarded, and the cell pellet was resuspended in binding buffer (500 mM NaCl, 50 mM Tris pH 8.5, 10 % glycerol, 2 mM sodium DT, 0.01% Tween 20, 50 mM arginine–glutamate and 20 mM imidazole) supplemented with 0.2 mg ml^−1^ bovine pancreatic deoxyribonuclease I and one cOmplete EDTA-free protease inhibitor tablet (Sigma-Aldrich) per 30 ml of pellet. Cells were lysed in a French press FA-078AE (Thermo Fisher Scientific) at 20,000 psi. The soluble fraction was separated by ultracentrifugation at 119.028*g* for 1 h at 4 °C, and filtered before loading onto a Strep-Tactin XT 4Flow high-capacity column (IBA Lifesciences) and a HisTrap HP column (Cytiva) connected to an ÄKTA pure chromatography system (Cytiva) inside an anaerobic COY tent. Binding buffer containing 50 mM biotin was used for the elution of Strep-tagged Mar(DK)_2_, while binding buffer containing 250 mM imidazole was used for the elution of His-tagged MarH_2_. Eluted proteins were buffer exchanged in Sephadex G-25 packed PD-10 desalting columns (Cytiva) to desalting buffer (150 mM NaCl, 50 mM Tris pH 7.8, 10% glycerol and 2 mM sodium DT), and concentrated using Amicons Ultra 15 ml filters (Merck) of 30 kDa molecular weight cut-off. Protein yields were determined using Quick Start Bradford 1× Dye Reagent (Bio-Rad Laboratories) following the manufacturer’s instructions. Purified protein was stored in liquid nitrogen until further use. The purity of the proteins was determined by sodium dodecyl sulfate–polyacrylamide gel electrophoresis (SDS–PAGE), the metal content by ICP-OES and the molecular mass by mass photometry as previously described^17^. In short, mass photometry measurements were carried out on microscope coverslips (1.5 H, 24 × 50 mm; Carl Roth) with CultureWell Reusable Gaskets (CW-50R-1.0, 50–3 mm diameter × 1 mm depth). Measurements were set up in gaskets assembled on microscope coverslips on the stage of a TwoMP mass photometer (Refeyn) with immersion oil. Samples were measured in anaerobic measurement buffer (150 mM NaCl, 50 mM Tris (pH 7.8), 10% glycerol and 10 mM sodium DT). Then, 0.5 µl of sample (500 nM stock concentration, 4 mM sodium DT) was removed from an anaerobic vial, quickly added to 19.5 µl of measurement buffer and mixed on the stage of the mass photometer. Measurements were started ~5 s after removing protein from the anaerobic environment. Data were acquired for 60 s at 100 frames per second using AcquireMP v2.3 (Refeyn). Mass photometry data sets were processed and analysed using DiscoverMP v20222 R1 (Refeyn).

### Homocitrate determination

Semi-quantitative determination of homocitrate was performed using high-resolution electrospray liquid chromatography–mass spectrometry. The chromatographic separation was performed on an Agilent Infinity II 1290 HPLC system (Agilent Technologies), using a Kinetex EVO C18 column (150 × 2.1 mm, 3 μm particle size, 100 Å pore size; Phenomenex) connected to a guard column of similar specificity (20 × 2.1 mm, 3 μm particle size; Phenomenex) at a constant flow rate of 0.2 ml min^−1^ with mobile phase A being 0.1% formic acid in water and phase B being 0.1% formic acid in methanol at 40 °C. The injection volume was 5 µl. The profile of the mobile phase consisted of the following steps and linear gradients: 0–0.5 min constant at 0% B; 0.5–6 min from 0% to 90% B; 6–7 min constant at 90% B; 7–7.1 min from 90% to 0% B; 7.1–12 min constant at 0% B.

An Agilent 6546 Q-TOF mass spectrometer (Agilent Technologies) was used in negative ionization mode with a dual electrospray ionization source: electrospray ionization spray voltage 2,000 V, nozzle voltage 500 V, sheath gas 300 °C at 11 l min^−1^, nebulizer pressure 45 psig and drying gas 170 °C, at 5 l min^−1^. The instrument was used in low molecule size mode with a screening window of 50–1,700 *m*/*z*. For online mass calibration, 112.9856 and 1033.9881 were used.

Compounds were identified on the basis of their accurate mass within a mass window of 5 ppm and their retention time compared with standards. Chromatograms were integrated using MassHunter software (Agilent). Relative abundance was determined based on the peak area by applying a mass extraction window of 10 ppm.

### Methylthio-alkane reductase in vitro activity assays

Enzymatic activity of methylthio-alkane reductase and Mo-nitrogenase was determined by in vitro activity assays with the substrates MT-EtOH, EMS, DMS, protons (under Ar) and acetylene (C_2_H_2_, 10% in Ar) measuring production of ethylene (C_2_H_4_), ethane (C_2_H_6_), methane (CH_4_), molecular hydrogen (H_2_) and C_2_H_4_ as well as C_2_H_6_, respectively. The reductase component (0.15 mg) was added to the catalytic component (0.1 mg) in 700 µl of reaction mix containing 50 mM Tris pH 7.8, 3.5 mM ATP, 7.87 mM MgCl_2_, 44.6 mM creatine phosphate, 0.2 mg ml^−1^ creatine phosphokinase, 5 mM DT and 15 mM of the corresponding VOSC in 1.2 atm Ar, if indicated. Due to the tendency of MarH_2_ to precipitate at high concentrations, all assays were conducted at a catalytic:reductase component molar ratio of 1:5. The reactions took place in 10-ml sealed gas chromatography (GC) vials, at 30 °C and shaking at 200 rpm for 10 min after the addition of the catalytic component. Samples were quenched with 300 µl of 400 mM Na_2_EDTA pH 8.0 up to a total volume of 1 ml. Content of the headspace was determined using a Clarus 690 GC system (PerkinElmer), as previously described^17^.

### NMR ^15^NH_4_ quantification

In vitro nitrogen fixation activity was determined for methylthio-alkane reductase and Mo-nitrogenase by running physiological assays as described above or Eu(II)-DTPA assays, which were performed in a 1.5-ml volume containing 5 mg of the catalytic component Mar(DK)_2_, 20 mM Eu(II)-DTPA, 25 mM Tris pH 8.0 and 2 mM DT. Both assays conditions were performed under 50% ^15^N_2_ (Merck) and 50% Ar at 2 atm in 10 ml GC vials, at 30 °C, shaking at 250 rpm for 5 h (physiological assays) or 15 h (Eu(II)-DTPA assays) quantifying ^15^NH_4_ with NMR. Negative controls were prepared under Ar or ^14^N_2_ atmospheres. Proteins were removed from the samples by ultrafiltration at 3,000*g* and 30 min using Amicon Ultra filters (3 kDa cut-off, Merck). Subsequently, 500 µl of filtrate were combined with 50 µl of 10 M H_2_SO_4_. Then, 50 µl of d6-DMSO was added as a locking agent to produce a final volume of 600 µl. Two reference samples of 30 mM NH_4_^+^ were prepared using natural abundance ^15^N-NH_4_Cl in assay buffers.

To quantify the concentration of ^14^NH_4_^+^ in each sample, one-dimensional ^1^H spectra with excitation sculpting water suppression were acquired using the zgesgp sequence from the Bruker standard sequence library on an Oxford Instruments 600 MHz magnet equipped with an Avance III console and a TXO Helium cooled cryoprobe. To quantify the concentration of ^15^NH_4_^+^, ^15^N heteronuclear single quantum coherence (HSQC) one-dimensional spectra were acquired on the same hardware using the hsqcetf3gp pulse sequence. Ninety-degree ^1^H pulse times were calibrated on water. The ^1^H carrier was set on-resonance at water to ensure optimal water suppression. The ^15^N carrier was set on-resonance for the ammonium signal at 21 ppm. The 90° ^15^N pulse time was calibrated on the high signal-to-noise Mo-nitrogenase sample and assumed constant for the other experiments where calibration was impossible due to low signal-to-noise ratios.

Spectra with varying numbers of scans were recorded for all samples dependent on the observable signal. The signal was rescaled linearly by the number of scans and the receiver gain. The spectra were then processed with zero-filling, an exponential window function and Fourier transformation using the nmrGlue python package^59^.

The ^15^NH_4_^+^ peak was then fitted in the ^15^N HSQC spectrum using a single Lorentzian function within the predefined grey window in Extended Data Fig. 7. The fitted peak was integrated and scaled against the reference peak to provide a ^15^NH_4_^+^ concentration. A region containing only noise was defined between 0 ppm and 1 ppm, and the standard deviation of this region was multiplied by the fitted full-width-at-half-maximum parameter to estimate the peak integral error.

The ^14^NH_4_^+^ peaks were fitted in the water-suppressed spectrum by first finding all local maxima above a spectrum-specific threshold, adding extra manual locations where needed, and fitting the resulting n Lorentzian functions to the data. The ^14^NH_4_^+^ triplet was identified within these fitted peak locations by identifying the characteristic ^14^N–^1^H coupling constant of 52.5 Hz. The integrals of the triplet were summed, and the fitting error was estimated as the standard deviation of the individual triplet peak integrals. These values were scaled against the respective buffer reference samples to provide a concentration of ^14^NH_4_^+^ within the sample.

Two sources of NH_4_^+^ signal were assumed: protein degradation and nitrogen fixation. All protein was produced in unenriched media, so it was assumed to contain a natural abundance 996:4 ratio of ^14^N and ^15^N. Therefore, we assumed that protein degradation contributes a natural abundance ratio of ^14^NH_4_^+^ and ^15^NH_4_^+^ molecules. To account for this, we subtracted a 4/996 fraction of each ^14^NH_4_^+^ concentration from the corresponding ^15^NH_4_^+^ concentration. The subtracted ^15^NH_4_^+^ signal was then used to calculate the total turnover number.

### Cryo-EM sample preparation, collection and processing

MgADP-AlF_3_-trapped Mar(DK)_2_(H_2_)_2_-complex was prepared by starting a 5-ml reaction with 22 nmol of MarH_2_ and 3 nmol of Mar(DK)_2_ in the presence of 100 mM MOPS pH 7.3, 50 mM Tris pH 7.8, 100 mM NaCl, 5 mM DT, 4 mM NaF, 0.2 mM AlCl_3_, 8 mM MgCl_2_ and 1 mM ATP. After 3 h incubation at 30 °C and 200 rpm, particles with the appropriate molecular mass were separated via size-exclusion chromatography in a HiLoad 26/600 Superdex 200 pg column (Cytiva) and buffer exchanged to 50 mM Tris pH 7.8, 200 mM NaCl and 5 mM DT. The protein complex with a measured molecular mass of ~368 kDa (Extended Data Fig. 2a,b; theoretical mass 344 kDa) was concentrated in Amicon Ultra filters (30 kDa cut-off, Merck) to 0.3 mg ml^−1^. Grids were prepared with a Vitrobot Mark IV (Thermo Fisher Scientific) placed inside an anaerobic COY vinyl tent (COY Laboratory Products) with a >95% N_2_ <5% H_2_ atmosphere, at 26 °C and 19% humidity. Four microlitres of sample were added to freshly glow-discharged 300 mesh 2/1 µm C-flat copper grids (Electron Microscopy Sciences) and blotted for 10 s at blot force 4 at 100% relative humidity and 4 °C before being plunge frozen in a liquid mix of 37% (v/v) ethane and 63% (v/v) propane.

Cryo-EM data collection was carried out on a Titan Krios G3i electron microscope (Thermo Fisher Scientific) operated at 300 keV in EFTEM mode and equipped with a BioQuantum-K3 imaging filter (Gatan). Data were collected in electron counting mode with EPU (version 3.6) software (Thermo Fisher Scientific) at a nominal magnification of 105,000×, corresponding to a calibrated pixel size of 0.837 Å per pixel, with a total dose of 50 e^−^ Å^−2^ per image, 50 fractions and 2.6 s exposure time. The energy filter slit width was set to 30 eV, and the defocus range was −1.0 µm to −2.5 µm at increments of 0.3 µm.

Cryo-EM data analysis was carried out in CryoSPARC v4.4.1 (ref. ^60^) (Extended Data Fig. 3). A total of 16,951 micrographs were subjected to patch motion correction and patch contrast transfer function estimation before a template picking using the projections of the Fe-nitrogenase cryo-EM volume as templates (EMD-16890)^17^. An initial set of 10,625,194 particles were extracted at a box size of 380 pixels and used for three consecutive rounds of 2D classification into 200 classes. Particles from the best 2D classes displaying features of at least one MarH_2_ bound to Mar(DK)_2_ were reextracted at a box size of 380 pixels, yielding a total of 3,209,215 particles. These were subjected to ab initio reconstruction into three classes. The best ab initio class with 1,286,797 particles was used for non-uniform refinement, yielding a global reconstruction of 2.45 Å with very poor features around both MarD subunits. Hard 3D classification with a local mask on the proximal MarD subunit and 5 Å target resolution resulted in 25 different classes, including classes completely or partially lacking MarD. Particles of the largest class (116,370 particles) displayed the best alignment with reduced particle orientation bias and were subjected to local refinement with a local mask on the proximal MarD and non-uniform refinement, yielding electron density maps with resolutions of 2.86 Å and 2.77 Å, respectively. The final electron density map was obtained by performing a local refinement using a solvent mask yielding a final resolution of 2.75 Å. The resulting map was autosharpened in Phenix v1.21.1 (ref. ^61^). Details on cryo-EM data collection, model building and refinement statistics can be found in Supplementary Table 1.

### Model building and refinement

The initial rigid-body fit of an AlphaFold2 (ref. ^62^) multimer model of the Mar(DK)_2_H_2_-complex was performed in ChimeraX-1.7.1 (ref. ^63^). Afterwards, manual fitting and refinement of all ligands was performed in Coot v0.8.9.2 (ref. ^64^) using the restraint files from the Coot ligand database (ADP, AF3 (AlF_3_), SF4 ([Fe_4_S_4_]-cluster), CLF (P-cluster) and S5Q ([Fe_8_S_9_C]-cluster)) allowing bond length deviation between 0.044 Å and 0.124 Å for the metalloclusters as specified in restraint files. Then, automatic model refinements were performed with phenix.real_space_refine of the Phenix software package (Phenix v1.21.1)^61^ using the same restrain files. For regions of the map that were not resolved well enough, the corresponding parts of the model were removed. Additional cycles of manual refinements in Coot as well as automatic refinements with phenix.real_space_refine were performed to obtain the final model, always applying ligand restraints.

### Substrate channel estimation

Substrate access channels to the active site of the methylthio-alkane reductase were calculated using the tool CAVER Analyst 2.0 BETA^36^. The starting point for channel calculating was the proposed binding site S2B of the [Fe_8_S_9_C]-cluster. The probe radius, shell radius and shell depth were set to 0.6, 3.0 and 4.0 Å, respectively. The two most likely substrate channels, with a maximum bottleneck radius of 27.5 Å and 25.6 Å, respectively, were selected for analysis (Fig. 4b,f).

### EPR spectroscopy

All EPR samples were prepared under anaerobic conditions in a Coy anaerobic glove box. All protein samples were buffer exchanged with Sephadex G-25 packed PD-10 desalting columns (Cytiva) into 500 mM NaCl, 50 mM Tris pH 8.5 and 10% glycerol to remove excess DT before sample preparation.

For the redox titrations, samples were titrated in the presence of a cocktail of 40 µM of each redox mediator through the addition of different volumes of buffered DT or potassium ferricyanide solutions (1.5–100 mM) until the desired redox potential was reached. The mediator mix consisted of 2,6-dichlorophenolindophenol (*E*_0_ = +217 mV), phenazine methosulfate (*E*_0_ = +55 mV), methylene blue (*E*_0_ = +11 mV), resorufin (*E*_0_ = −51 mV), IDS (*E*_0_ = −125 mV), 2-hydroxy-1,4-napthaquinone (*E*_0_ = −152 mV), sodium anthraquinone 2-sulfonate (*E*_0_ = −225 mV), phenosafranin (*E*_0_ = −252 mV), safranin O (*E*_0_ = −289 mV), neutral red (*E*_0_ = −329 mV), benzyl viologen (*E*_0_ = −358 mV) and methyl viologen (*E*_0_ = −449 mV). Upon stabilization of the redox potential, a 300-µl sample was withdrawn, transferred to an EPR quartz tube, capped and shock frozen in liquid nitrogen. The InLab Argenthal microelectrode (Ag/AgCl, *E* = +207 mV versus H_2_/H^+^, with combined Pt counter electrode) was calibrated with a quinhydrone saturated pH 7 reference buffer solution (*E* = +285 mV versus H_2_/H^+^ at 25 °C).

MarH_2_ samples were prepared by gentle addition of anaerobic stock solutions of the compounds of interest (2 mM DT, 5 mM MgATP, final concentration) to the protein, incubated for at least 3 min and frozen as for the redox titration. For the turnover experiment Mar(DK)_2_ was oxidized by addition of 2 equiv. IDS and incubated for 15 min. DT-reduced MarH_2_ and the IDS-oxidized Mar(DK)_2_ were desalted. Directly after desalting, 300-µl samples of MarH_2_ and oxidized and Mar(DK)_2_ were frozen separately. DT-removed MarH_2_ and IDS-removed Mar(DK)_2_ were mixed in ratio of 1.2:1 reductase:catalytic component with MgATP (5 mM final concentration) incubated for 1 min, concentrated with an Amicon Ultra filter (100 kDa cut-off, Merck) to a final volume of 300 µl and shock frozen. Nif(DK)_2_ was purified as described^18^, desalted and treated with 2 mM DT, 0.5 mM IDS or 50 µM IDS.

Cw-EPR spectra were recorded with a commercial Bruker spectrometer composed of an X-band E580-10/12 bridge and a 4122HQE resonator, for regular (perpendicular mode, for Fig. 3 and Extended Data Fig. 8) and an ER4116 dual mode cavity for parallel mode measurements (for Nif(DK)_2_ and trials to detect *g* = 12 or *g* = 16 signals in Mar(DK)_2_) measurements. The system was equipped with an Oxford Instruments temperature controller and ESR 900 cryostat, which was cryocooled by a Stinger (Cold Edge Technologies) linked to helium compressor (Sumitomo F-70). Spectral simulations were performed with GeeStrain5 (ref. ^65^) or in Excel (Gaussian curves representing the absorption-shaped highest *g*-value of the very anisostropic doublets of the *S* = 3/2 system of MarH_2_, or the first derivative of a Gaussian curve for the isotropic *g* = 4.3 EPR signal of the middle doublet of high spin ferric iron). Except for Fig. 3f and Extended Data Fig. 9, five-point moving averages were used to limit the noise of EPR spectra.

### Phylogenetic analysis and ancestor prediction

For the phylogeny in Extended Data Fig. 10, sequences from *Methanolobus bombayensis* Ni^2+^-sirohydrochlorin *a*,*c*-diamide reductive cyclase complex component D (CfbD, WP_209620388.1), *R. rubrum* methylthio-alkane reductase MarDK operon (MarD, WP_011388552.1; MarK WP_011388551.1), *A. vinelandii* nitrogenase NifDKEN operon (NifD, WP_012698832.1; NifK, WP_012698833.1; NifE, WP_012698838.1; NifN, WP_012698839.1), *R. capsulatus* chlorophyllide *a* oxidoreductase BchYZ operon (BchY, WP_023914767.1; BchZ WP_023914765.1) and DPOR BchNB operon (BchN, WP_023913723.1; BchB WP_013066408.1) were used as queries across major archaeal and bacterial lineages, collecting a total of 996 sequences using the web interface of NCBI pblast. Sequences were collected iteratively between 2021 and 2024, and filtered to retain only those with E-values ≤1 × 10^−30^ and query coverage >70%. Sequences were initially assigned as bona fide nitrogenase components through synteny, for cases where NifDKEN are in synteny, because operons of other relatives of this family contain only DK paralogues. Further classification during dataset assembly was achieved through reciprocal best-blast hits. Ultimately, the classification on our tree is based on branching relationships of known sequences in each clade. Sequences were aligned using Muscle v5 (ref. ^66^) and further refined using clipKIT^67^. We have also realigned our sequences three times each with two different algorithms (geneafpair and globalpair algorithms with option -maxiterate 3000) in MAFFT, and verified that the alignment is robust—specifically, that all reported cysteines are consistently aligned in each case. The resulting phylogenetic trees were constructed using Iqtree2 (ref. ^68^) under the LG + G4 model, with 35,000 ultrafast bootstraps and 1500 SH-aLRT replicates^69^. Model selection for evolution was based on the corrected Akaike information criterion (-m MFP -merit AICc option in Iqtree2). The resulting tree was constrained to have BchY and BchN sister to NifDE and nitrogenase-like proteins (NflD) including MarD, and BchB and BchZ clades sister to NifKN, NflK and MarK. Every other node was polytomized. The resulting maximum likelihood analysis of the constrained tree shows similar likelihood values as for the unconstrained tree and it was not rejected by any statistical tree topology test performed with iqtree. This topology was chosen for analysis owing to the more parsimonious history in terms of gene duplication: all nitrogenase relatives other than CfbD are heterotetramers made from two paralogues. In our constrained topology, the tree implies that a single gene duplication of an ancestral homomer produced this arrangement. Subsequent operon duplications would then produce the different types of nitrogenase-like complex, each also consisting of two paralogues (including the maturases of nitrogenases). In the unconstrained tree, all BchNBYZ paralogues form a monophyletic sister group to all other nitrogenase-like genes. This would imply that Bch separately underwent a duplication of an ancestral homomer, which then underwent an operon duplication to yield BchNB and BchYZ. This implies one additional gene duplication compared with our constrained tree and, thus, less parsimony. Supporting values for this topology were calculated with 500 Felsenstein bootstrap analysis using Iqtree2 and the approximate likelihood ratio test (aLRT) using PhyML. Ancestral sequence reconstruction for the nodes of interest was performed using a wrapper script that uses Iqtree2 for ancestral sequence inference using the LG + G4 model and PAUPv4 for a Fitch parsimony inference of which sites should be gaps in our alignment.

### AlphaFold Structure Prediction

The structure of the last common nitrogenase and methylthio-alkane reductase ancestor AncD and AncK was predicted by AlphaFold 2^62^ using ColabFold^70^ with default parameters (without templates, model_type: alphafold2_multimer_v3, num_relax: 1).

### Reporting summary

Further information on research design is available in the Nature Portfolio Reporting Summary linked to this article.

## Supplementary information

Supplementary Information Supplementary Discussions 1–4, Tables 1–6 and references.

Reporting Summary

## Data Availability

The refined MarDK_2_H_2_ structure and electron density map were deposited under PDB-ID 9FMG and EMD-50553, respectively. Raw data of SDS–PAGE analysis, ICP-OES and mass photometry measurements, gas chromatography and NMR enzyme activity assays, EPR spectroscopy, protein sequences used in multiple sequence alignments, and phylogenetic analysis (protein sequences identifiers, multiple sequence alignment, uncollapsed trees with bootstrap values, and resurrected ancestral protein sequences with calculated posterior probabilities per site) are deposited on Edmond, the Open Research Data Repository of the Max Planck Society (10.17617/3.OHDUAZ)^71^, which will be released upon acceptance.

## Code availability

All NMR data and the Python package created for analysis are available via GitHub at https://github.com/charliebuchanan/nitrogenaseNMR.
