# Supplementary material for: Methylthio-alkane reductases use nitrogenase metalloclusters for carbon–sulfur bond cleavage
Source: Nat Catal. 2025 Oct 23;8(10):1086–99. doi: 10.1038/s41929-025-01426-2 (PMC12552129; doi:10.1038/s41929-025-01426-2)
Supplement: Supplementary file 1 — Supplementary Discussions 1–4, Tables 1–6 and references. [file 41929_2025_1426_MOESM1_ESM.pdf]

nature catalysis

Supplementary information

https://doi.org/10.1038/s41929-025-01426-2

Methylthio-alkane reductases use nitrogenase metalloclusters for carbon–sulfur bond cleavage

In the format provided by the authors and unedited

The PDF file includes:

Supplementary Discussion 1 to 4
Supplementary Tables 1 to 6
References

Supplementary Discussion

Supplementary Discussion 1: The reductase component $MarH_2$ belongs to the family of P-loop NTPases

Analyzing the structure, we observed a canonical reductase component $MarH_2$ containing switch I and switch II regions, which facilitate conformational changes associated with nucleotide binding and hydrolysis [1] (Extended Data Fig. 4a). Each monomer binds a MgADP-AlF_3 moiety at the Walker A motif (GKGGIGKS) [2], indicating the ability of $MarH_2$ to bind and hydrolyze MgATP (Fig. 3b and Extended Data Fig. 4b). $MarH_2$ coordinates a single [Fe_4S_4]-cluster in its dimeric interface by Cys97^{MarH} and Cys133^{MarH} (Fig. 3c).

Supplementary Discussion 2: Comparison of P-cluster surroundings in Mo-nitrogenase and methylthioalkane reductase

The P-cluster of *A. vinelandii*'s Mo-nitrogenase undergoes structural rearrangements upon oxidation [3]. In the one-electron oxidized P^{1+} state, the nitrogenase P-cluster symmetry is disrupted by the binding of the conserved Ser188$^{\mathit{Av}\mathrm{NifK}}$ to Fe6 [4], while an additional coordination of Fe5 by the backbone amide of Cys88$^{\mathit{Av}\mathrm{NifD}}$ occurs at the two-electron oxidized P^{2+} state [5]. In the Mo-nitrogenase of *Gluconacetobacter diazotrophicus*, the serine is substituted by an unusual coordination between Tyr98$^{\mathit{Gd}\mathrm{NifK}}$ and Fe8 [6]. While MarK lacks structural analogs of both Ser188$^{\mathit{Av}\mathrm{NifK}}$ and Tyr98$^{\mathit{Gd}\mathrm{NifK}}$, Cys52^{MarK} or Gln60^{MarK} might coordinate Fe8, akin to Tyr98$^{\mathit{Gd}\mathrm{NifK}}$ (Extended Data Fig. 6b). Alternatively, P-cluster coordination upon oxidation could involve Fe4 and the residues Ser157^{MarD} or Glu68^{MarD}. However, in the current structure all these potential ligands are at relatively long distances of 4.8-5.7 Å to the respective Fe atoms. Our findings strongly suggest that the P-cluster of methylthio-alkane reductase is in the reduced state (P^N), however, future studies on different redox states will be necessary.

Supplementary Discussion 3: EPR features of $MarH_2$

The temperature dependency of the two absorption-shaped peaks at g=5.79 and g=5.03 of the $[Fe_4S_4]^{1+}$ cluster S=3/2 state define the S=3/2 spin Hamiltonian parameters as |E/D|=0.20 and D≈ 2 cm^{-1} in the absence of ATP (Extended Data Fig. 8d-g, and Extended Data Table 2). Structural changes induced by ATP decrease the S=1/2 content and the amplitude of the zero-field splitting parameter D of the S=3/2 species in the $MarH_2$ homodimer, in agreement with its function as ATP-dependent reductase.

Supplementary Discussion 4: Differences between EPR features of $Mar(DK)_2$ and $Nif(DK)_2$

We employed the catalytic component of the Mo-nitrogenase $Nif(DK)_2$ from *R. capsulatus* as EPR spectroscopic benchmark (Extended Data Fig. 9 and Extended Data Table 3). Though S=3/2 EPR signals of FeMoco in the DT-reduced state, a mixture of two very intense S=1/2 signals of the P^{1+}-cluster and the g=15.7 signal of the P^{2+}-cluster, were easily detected for $Nif(DK)_2$, no such signals were found for $Mar(DK)_2$ in various redox states. Although both methylthio-alkane reductase and nitrogenase contain a P-cluster, their electron transport mechanisms may differ since the characteristic P^{2+} integer spin EPR signal [7] could not be detected. These differences likely originate from dissimilarities in the protein environments. The methylthio-alkane reductase lacks the conserved Ser188$^{\mathit{Av}\mathrm{NifK}}$ of nitrogenases, which is involved in P-cluster ligation upon oxidation.

Supplementary Table 1: Information of cryo-EM data collection and model refinement

	**MarDK$_2$H$_2$ (EMD-50553), (PDB: 9FMG)**
**Data collection and processing**	
Magnification	105,000
Voltage (keV)	300
Electron exposure (e^{-}/Å^2)	50
Defocus range (μm)	−1.0 to −2.5
Pixel size (Å)	0.837
Symmetry imposed	C1
Initial particle images (no.)	10,625,194
Final particle images (no.)	116,370
Map resolution (Å)	2.75 / 4.10 (masked / unmasked)
FSC threshold	0.143
Map resolution range (Å)	1.8–12.4
Map sharpening B factor (Å^2)	-71.5
**Refinement**	
Initial model used	AlphaFold 2
Model resolution (Å)	2.71 / 2.74 (masked / unmasked)
FSC threshold	0.143
CC_{Map}	0.76
Model composition	
Non-hydrogen atoms	13,763
Protein residues	1,768
Ligands	2 × ADP, 2 x AlF_3, 2 × Mg^{2+}, 1 × [Fe_8S_9C], 1 × [Fe_8S_7], 1 × [Fe_4S_4]
B factors (Å^2)	
Protein (min / max / mean)	15.55 / 108.68 / 42.94
Ligand (min / max / mean)	13.40 / 63.17 / 38.37
R.m.s. deviations	
Bond lengths (Å)	0.28
Bond angles (°)	0.51
**Validation**	
MolProbity score	1.71
Clashscore	6.00
CaBLAM outliers	2.53
Poor rotamers (%)	0.28
C-beta deviations	0.00
Ramachandran plot	
Favored (%)	94.47
Allowed (%)	5.53
Disallowed (%)	0.00

Supplementary Table 2: Parameters for the simulation of *S*=3/2 EPR spectra of $MarH_2$

EPR species	T (K)	*g*-value	Ampli-tude	FWHM*	*g*-value	Ampli-tude	FWHM*	*g*-value	Ampli-tude†	FWHM*†
$MarH_2$ without ATP	4.0	5.79	0.50	7.0	5.03	0.45	32	4.29	4.5	5.5
	10	5.79	0.18	7.0	5.03	0.42	32	4.29	5.0	5.5
	20	5.79	0.05	7.0	5.03	0.21	32	4.29	3.0	5.5
$MarH_2$ with ATP	4.0	5.76 ‡	nd §	10	5.06	1.07	28	4.28	7.0	5.5
	10	5.76 ‡	nd §	10	5.06	0.62	28	4.28	6.5	5.5
	20	5.76 ‡	nd §	10	5.06	0.23	28	4.28	4.0	5.5

* Full width at half maximum (FWHM) of the simulation with a Gaussian line shape.

† Amplitude and FWHM refer to the simulation before calculation of its derivative.

‡ Calculated *g*-value from the rhombogram using *g*=5.06 (see Extended Data Fig. 8).

§ Not detectable, possibly too broad.

Supplementary Table 3: Parameters for the simulation with GeeStrain5 of *S*=1/2 EPR spectra

EPR species	g_{av}	g_x	g_y	g_z	W_{xx}	W_{yy}	W_{zz}	w_{xy}	w_{xz}	w_{yz}
Low potential*	1.959	1.880	1.936	2.060	0.030	0.010	0.030	0.007	0.025	0
L-cluster-like†	1.908	1.830	1.926	1.967	0.095	0.041	0.022	0	0	0.006
P^{1+} major‡	1.955	1.841	1.962	2.062	0.008	0.006	0.007	0	0	0
P^{1+} minor‡	1.933	1.885	1.907	2.006	0.006	0.010	0.005	0	0	0

* For reduced $Mar(DK)_2$ in Fig. 3g (bottom trace).

† For oxidized $Mar(DK)_2$ in Fig. 3g (trace below $Nif(EN)_2$).

‡ For 0.5 mM IDS oxidized $Nif(DK)_2$ of *R. capsulatus* in Extended Data Fig. 9c. Ratio major to minor species: 1 to 0.20 (in GeeStrain5, corresponding to integrated intensity).

Supplementary Table 4: Strains used in this study

Strain	Genotype	Reference
Rhodospirillum rubrum ATCC 11170 / S1	Wildtype	Ref. [8]
Rhodobacter capsulatus B10S	Wildtype	Ref. [9]
Rhodobacter capsulatus MM0246	*ΔnifD::SpR ΔanfHDGK::gmR ΔdraTG ΔmodABC ΔgtaI*	Ref. [10]
Rhodobacter capsulatus MM0422	*ΔnifD::SpR ΔanfHDGK::gmR ΔdraTG ΔmodABC ΔgtaI* / pMM0181	This study
Rhodobacter capsulatus MM0468	*ΔnifHDK ΔanfHDGK::GmR ΔdraTG ΔgtaI*	Ref. [11]
Rhodobacter capsulatus MM0480	*ΔnifHDK ΔanfHDGK::GmR ΔdraTG ΔgtaI* / pMM0207	Ref. [11]
Escherichia coli DH5α	F^- Φ80*lac*ZΔM15 Δ(*lac*ZYA-*arg*F) U169 *rec*A1 *end*A1 *hsd*R17(r_k^-, m_k^+) *pho*A *sup*E44 *thi*-1 *gyr*A96 *rel*A1 λ^-	Thermo Fisher Scientific, (Waltham, Massachusetts, USA) catalogue #18265017
Escherichia coli ST18	*pro thi hsdR*$^+$ Tp^r Sm^r; chromosome::RP4-2 Tc::Mu-Kan::Tn7/lpir *ΔhemA*	Ref. [12]

Supplementary Table 5: Primers used in this study

Primer	Sequence	Purpose
oMM0494	CACCACAGGTCTCGTATGACGGTTCCTGCTTATCCTTCC	Construction of pMM0165
oMM0495	CACCACAGGTCTCGAGCGTCAAGCGCTTGCGCTGA	Construction of pMM0165
oMM0649	CGCTCTTGGACTCCTG	Construction of pMM0170 and pMM0181
oMM0650	GTTAAACAAAATTATTTCTAGACGGC	Construction of pMM0170 and pMM0181
oMM0651	GGCCGTCTAGAAATAATTTTG	Construction of pMM0181
oMM0652	CTGGATCTATCAACAGGAGTC	Construction of pMM0170 and pMM0181
oMM0662	ATGGTGATGATGGTGGTGCATGGTTCTCTCCGTC	Construction of pMM0181
oMM0663	CACCACCATCATCACCATGCCAAAAGTCCCAAAC	Construction of pMM0181
oMM0668	CACCTGGCCGTCTAGAAATAATTTTG	Construction of pMM0170
oMM0669	TTTTTCGAACTGCGGGTGGCTCCACTCTGCGGGACGGCG	Construction of pMM0170
oMM0670	TGGAGCCACCCGCAGTTCGAAAAATGAGCCCCGTCATGC	Construction of pMM0170

Supplementary Table 6: Plasmids used in this study

Plasmid	Code	Description	Reference
pOGG024	pMM0114	Broad-host range and medium copy number, pBBR1 with *oriT*, *lacZα* cassette for golden gate cloning (BsaI), Gen^R	Ref. [13]
pOGG024-*kanR*	pMM0119	Broad-host range and medium copy number, pBBR1 with *oriT*, *lacZα* cassette for golden gate cloning (BsaI), Kan^R	Ref. [10]
pOGG024-*kanR*	pMM0129	Broad-host range and medium copy number, pBBR1 with *oriT*, *lacZα* cassette for golden gate cloning (BsaI), *anfH* promoter, Kan^R	Ref. [14]
pOGG024-*kanR marBHDK*	pMM0165	Broad-host range and medium copy number, pBBR1 with *oriT*, *marBHDK* cloned into BsaI site, *anfH* promoter, Kan^R	This study
pOGG024-*kanR marBHDK*-Strep	pMM0170	Broad-host range and medium copy number, pBBR1 with *oriT*, *marBHDK* cloned into BsaI site with C-terminal Strep tag II on *marD*, *anfH* promoter, Kan^R	This study
pOGG024-*kanR marBHDK*-Strep/His	pMM0181	Broad-host range and medium copy number, pBBR1 with *oriT*, *marBHDK* cloned into BsaI site with N-terminal hexahistidine tag on *marH* and C-terminal Strep tag II on *marD*, *anfH* promoter, Kan^R	This study
pOGG024-*kanR nifHDK*-Strep/His	pMM0207	Broad-host range and medium copy number, pBBR1 with *oriT*, *nifHDK* cloned into BsaI site with N-terminal hexahistidine tag on *nifH* and C-terminal Strep tag II on *nifD*, *nifH* promoter, Kan^R	Ref. [11]

References

1. Georgiadis, M. M. *et al.* Crystallographic structure of the nitrogenase iron protein from Azotobacter vinelandii. *Science* **257**, 1653–1659 (1992).
2. Walker, J. E., Saraste, M., Runswick, M. J. & Gay, N. J. Distantly related sequences in the alpha- and beta-subunits of ATP synthase, myosin, kinases and other ATP-requiring enzymes and a common nucleotide binding fold. *EMBO J.* **1**, 945–951 (1982).
3. Einsle, O. & Rees, D. C. Structural enzymology of nitrogenase enzymes. *Chem. Rev.* **120**, 4969–5004 (2020).
4. Keable, S. M. *et al.* Structural characterization of the P1+ intermediate state of the P-cluster of nitrogenase. *J. Biol. Chem.* **293**, 9629–9635 (2018).
5. Peters, J. W. *et al.* Redox-dependent structural changes in the nitrogenase P-cluster. *Biochemistry* **36**, 1181–1187 (1997).
6. Owens, C. P., Katz, F. E. H., Carter, C. H., Oswald, V. F. & Tezcan, F. A. Tyrosine-coordinated P-Cluster in G. diazotrophicus nitrogenase: evidence for the importance of O-based ligands in conformationally gated electron transfer. *J. Am. Chem. Soc.* **138**, 10124–10127 (2016).
7. Lee, C. C., Górecki, K., Stang, M., Ribbe, M. W. & Hu, Y. Cofactor maturase NifEN: A prototype ancient nitrogenase? *Sci. Adv.* **10**, eado6169 (2024).
8. Christine Munk, A. *et al.* Complete genome sequence of Rhodospirillum rubrum type strain (S1 T). *Stand. Genomic Sci.* **4**, 293–302 (2011).
9. Klipp, W., Masepohl, B. & Pühler, A. Identification and mapping of nitrogen fixation genes of Rhodobacter capsulatus: duplication of a nifA-nifB region. *J. Bacteriol.* **170**, 693–699 (1988).
10. Schmidt, F. V *et al.* Structural insights into the iron nitrogenase complex. *Nat. Struct. Mol. Biol.* **31**, 150–158 (2024).
11. Oehlmann, N. N., Schmidt, F. V., Herzog, M., Goldman, A. L. & Rebelein, J. G. The iron nitrogenase reduces carbon dioxide to formate and methane under physiological conditions: A route to feedstock chemicals. *Sci. Adv.* **10**, eado7729 (2024).
12. Thoma, S. & Schobert, M. An improved Escherichia Coli donor strain for diparental mating. *FEMS Microbiol. Lett.* **294**, 127–132 (2009).
13. Geddes, B. A., Mendoza-Suárez, M. A. & Poole, P. S. A Bacterial Expression Vector Archive (BEVA) for flexible modular assembly of golden gate-compatible vectors. *Front. Microbiol.* **9**, 3345 (2019).
14. Addison, H. *et al.* Two distinct ferredoxins are essential for nitrogen fixation by the iron nitrogenase in Rhodobacter capsulatus. *MBio* **15**, e0331423 (2024).
