# Supplementary material for: Methylthio-alkane reductases use nitrogenase metalloclusters for carbon–sulfur bond cleavage
Source: Nat Catal. 2025 Oct 23;8(10):1086–99. doi: 10.1038/s41929-025-01426-2 (PMC12552129; doi:10.1038/s41929-025-01426-2)
Supplement: Supplementary file 2 — Reporting Summary [file 41929_2025_1426_MOESM2_ESM.pdf]

nature portfolio

Corresponding author(s): Johannes G. Rebelein

Last updated by author(s): Jul 22, 2025

Reporting Summary

Nature Portfolio wishes to improve the reproducibility of the work that we publish. This form provides structure for consistency and transparency in reporting. For further information on Nature Portfolio policies, see our Editorial Policies and the Editorial Policy Checklist.

Statistics

For all statistical analyses, confirm that the following items are present in the figure legend, table legend, main text, or Methods section.

n/a	Confirmed	
☐	☒	The exact sample size (*n*) for each experimental group/condition, given as a discrete number and unit of measurement
☐	☒	A statement on whether measurements were taken from distinct samples or whether the same sample was measured repeatedly
☒	☐	The statistical test(s) used AND whether they are one- or two-sided<br>*Only common tests should be described solely by name; describe more complex techniques in the Methods section.*
☒	☐	A description of all covariates tested
☒	☐	A description of any assumptions or corrections, such as tests of normality and adjustment for multiple comparisons
☐	☒	A full description of the statistical parameters including central tendency (e.g. means) or other basic estimates (e.g. regression coefficient) AND variation (e.g. standard deviation) or associated estimates of uncertainty (e.g. confidence intervals)
☒	☐	For null hypothesis testing, the test statistic (e.g. *F*, *t*, *r*) with confidence intervals, effect sizes, degrees of freedom and *P* value noted<br>*Give P values as exact values whenever suitable.*
☒	☐	For Bayesian analysis, information on the choice of priors and Markov chain Monte Carlo settings
☒	☐	For hierarchical and complex designs, identification of the appropriate level for tests and full reporting of outcomes
☒	☐	Estimates of effect sizes (e.g. Cohen's *d*, Pearson's *r*), indicating how they were calculated

Our web collection on statistics for biologists contains articles on many of the points above.

Software and code

Policy information about availability of computer code

Data collection	TotalChrom v. 6.3.4 (PerkinElmer), AcquireMP v. 2.3 (Refeyn Ltd), ICP Expert Software v. 4.1.0 (Agilent), EPU software v. 3.6 (Thermo Scientific), MassHunter software (Agilent)
Data analysis	ChimeraX-1.7.1, AlphaFold 2 (Google DeepMind), CryoSPARC v4.4.1, Coot v0.8.9.2, Phenix v1.21.1, CAVER Analyst 2.0 BETA, PyMOL v.2.5.7, GeeStrain5, Excel, Muscle v5, clipKIT, Iqtree2, PhyML, PAUPv4, ColabFold, GraphPad Prism 10, DiscoverMP v. 2022 R1 (Refeyn Ltd), nmrGlue python package

For manuscripts utilizing custom algorithms or software that are central to the research but not yet described in published literature, software must be made available to editors and reviewers. We strongly encourage code deposition in a community repository (e.g. GitHub). See the Nature Portfolio guidelines for submitting code & software for further information.

Data

Policy information about availability of data

All manuscripts must include a data availability statement. This statement should provide the following information, where applicable:

- Accession codes, unique identifiers, or web links for publicly available datasets
- A description of any restrictions on data availability
- For clinical datasets or third party data, please ensure that the statement adheres to our policy

The refined MarDK2H2 structure and electron density map were deposited under PDB-ID 9FMG and EMD-50553, respectively. Raw data of SDS-PAGE analysis, ICP-

OES and mass photometry measurements, gas chromatography and NMR enzyme activity assays, EPR spectroscopy and protein sequences used in multiple sequence alignments are deposited on Edmond, the Open Research Data Repository of the Max Planck Society (https://doi.org/10.17617/3.OHDUAZ), which will be released upon acceptance.
All NMR data and the python package created for analysis have been uploaded to GitHub and can be found here: https://github.com/charliebuchanan/nitrogenaseNMR.

Research involving human participants, their data, or biological material

Policy information about studies with human participants or human data. See also policy information about sex, gender (identity/presentation), and sexual orientation and race, ethnicity and racism.

Reporting on sex and gender	n/a
Reporting on race, ethnicity, or other socially relevant groupings	n/a
Population characteristics	n/a
Recruitment	n/a
Ethics oversight	n/a

Note that full information on the approval of the study protocol must also be provided in the manuscript.

Field-specific reporting

Please select the one below that is the best fit for your research. If you are not sure, read the appropriate sections before making your selection.

☒ Life sciences ☐ Behavioural & social sciences ☐ Ecological, evolutionary & environmental sciences

For a reference copy of the document with all sections, see nature.com/documents/nr-reporting-summary-flat.pdf

Life sciences study design

All studies must disclose on these points even when the disclosure is negative.

Sample size	A sample size of n=3 (independent experiments) was used for gas chromatography activity assays according to common scientific practice to ensure reliability of results. A sample size of n=6 was used for the detection of homocitrate via HPLC-MS and for ICP-OES samples, 2 technical replicates of 3 biological replicates were used to ensure robustness of the assays across independent protein preparations. A sample size of n=2 (independent experiments) for the detection of 15NH4+ by NMR in the 15N2 reduction assays was deemed sufficient due to the high sensitivity of the technique.
Data exclusions	No data was excluded.
Replication	Most experiments were performed in replicates (n=2, n=3 or n=6) to ensure reproducibility of results. The number of replicates per sample are specified in the figure legends for each experiment and shown as individual data points. Mass photometry, EPR spectroscopy and single particle cryo-EM experiments were not performed in replicates due to the robustness of these techniques based on the analysis of protein particles dispersed in solution.
Randomization	For cryo-EM 3D refinement, two half maps were generated by randomly splitting all particles into two groups. No further randomization was necessary for this study.
Blinding	Blinding was not necessary for this study because no group allocation was involved.

Reporting for specific materials, systems and methods

We require information from authors about some types of materials, experimental systems and methods used in many studies. Here, indicate whether each material, system or method listed is relevant to your study. If you are not sure if a list item applies to your research, read the appropriate section before selecting a response.

Materials & experimental systems

n/a	Involved in the study
☒	☐ Antibodies
☒	☐ Eukaryotic cell lines
☒	☐ Palaeontology and archaeology
☒	☐ Animals and other organisms
☒	☐ Clinical data
☒	☐ Dual use research of concern
☒	☐ Plants

Methods

n/a	Involved in the study
☒	☐ ChIP-seq
☒	☐ Flow cytometry
☒	☐ MRI-based neuroimaging

Plants

Seed stocks

Report on the source of all seed stocks or other plant material used. If applicable, state the seed stock centre and catalogue number. If plant specimens were collected from the field, describe the collection location, date and sampling procedures.

Novel plant genotypes

Describe the methods by which all novel plant genotypes were produced. This includes those generated by transgenic approaches, gene editing, chemical/radiation-based mutagenesis and hybridization. For transgenic lines, describe the transformation method, the number of independent lines analyzed and the generation upon which experiments were performed. For gene-edited lines, describe the editor used, the endogenous sequence targeted for editing, the targeting guide RNA sequence (if applicable) and how the editor was applied.

Authentication

Describe any authentication procedures for each seed stock used or novel genotype generated. Describe any experiments used to assess the effect of a mutation and, where applicable, how potential secondary effects (e.g. second site T-DNA insertions, mosiacism, off-target gene editing) were examined.
